# Magnetomechanical Detachment of Bacterial Biofilms Using Anisotropic Magnetic Iron Oxide Nanochains

**DOI:** 10.1021/acsabm.5c01029

**Published:** 2025-09-04

**Authors:** Matija Šavli, Manca Černila, Maja Caf, Abida Zahirović, Nika Zaveršek, Sebastjan Nemec, Spase Stojanov, Anja Klančnik, Jerica Sabotič, Slavko Kralj, Aleš Berlec

**Affiliations:** † Department of Biotechnology, 61790 Jožef Stefan Institute, Jamova 39, Ljubljana 1000, Slovenia; ‡ Department for Materials Synthesis, 61790 Jožef Stefan Institute, Jamova 39, Ljubljana 1000, Slovenia; § Faculty of Pharmacy, University of Ljubljana, Aškerčeva 7, Ljubljana 1000, Slovenia; ∥ Department of Food Science and Technology, Biotechnical Faculty, University of Ljubljana, Jamnikarjeva 101, Ljubljana 1000, Slovenia

**Keywords:** iron oxide nanoparticles, magnetic nanoparticles, nanochains, bacteria, biofilm removal

## Abstract

Bacterial biofilms attach to various surfaces and represent an important clinical and public health problem, as they are highly recalcitrant and are often associated with chronic, nonhealing diseases and healthcare-associated infections. Antibacterial agents are often not sufficient for their elimination and have to be combined with mechanical removal. Mechanical forces can be generated by actuating nonspherical (anisotropic) magnetically responsive nanoparticles in a rotating magnetic field. We have thus prepared anisotropic superparamagnetic nanochains in the size range of 0.5–1 μm by magnetically assembling several iron oxide nanoparticle clusters and coating them with a layer of silica with different shell morphologies: smooth, moderately rough, and highly rough. The silica surface was additionally functionalized with carboxylic groups to increase colloidal stability. The efficacy of the nanochains in biofilm removal was studied systematically with three different model nonpathogenic bacterial species *Escherichia coli*, *Lactococcus lactis*, and *Pseudomonas fragi*; two different magnetic field strengths; two stirring speeds; and two treatment durations. All bacterial species were engineered to express fluorescent proteins to enable quantification of biofilm removal by colony-forming unit count and fluorescence measurements. Nanochains removed >90% of Gram-negative *E. coli* and *P. fragi* with a stronger magnetic field, and <90% of Gram-positive *L. lactis* with a weaker magnetic field. Surface roughness of nanochains, duration, and stirring speed also affected removal, but the effect could not be generalized. In contrast to their effects on biofilms, the functionalized nanochains showed no toxicity to Caco-2 intestinal epithelial cells, regardless of whether magnetomechanical force was employed or not. In summary, we demonstrated that remotely controlled spatial movement of nanoparticles can generate sufficient mechanical forces to disperse attached biofilms while retaining safety in an epithelial cell model.

## Graphical abstract

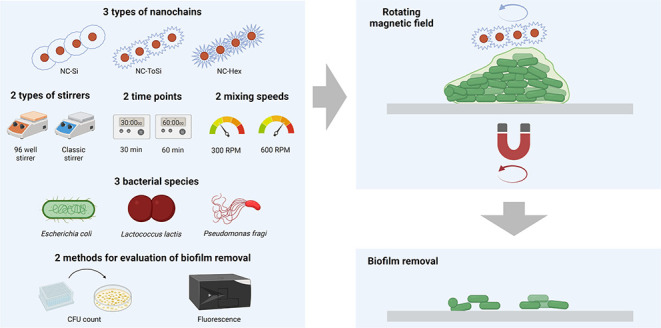

## Introduction

Biofilms are highly organized communities of microbes bound by an extracellular matrix, which is composed of extracellular polymeric substances (EPS) produced by the constituent bacterial cells. The polymeric substances include polysaccharides, proteins, nucleic acids, lipids, and other biomolecules that form a protective and adhesive layer around the microbes.^1^ Biofilms enable bacteria to survive and persist in environments by providing numerous advantages, such as water retention, nutrient storage, and physical protection from the host immune system and environmental factors.^2^ Biofilms form after bacteria attach to a surface through nonspecific interactions. The EPS surround the bacterial cells, provide structural support, and facilitate intercellular communication for the formation of the mature biofilm.^3^ Biofilms represent an important clinical and public health problem, and it has been estimated that up to 80% of all bacterial infections in humans are biofilm-associated.^4^ Such infections are highly recalcitrant and often associated with chronic, nonhealing diseases. Biofilms are also commonly associated with healthcare-associated infections (e.g., biofilms on medical indwelling devices^5^), which affect 3.8 million people in the EU each year and cause an estimated 90 000 deaths annually.^6^ Antibiofilm approaches often require a combination of antibacterial agents and mechanical removal, which is usually limited to surgical debridement, e.g., in chronic wounds.^7,8^

Metal and metal oxide nanoparticles exert their antibacterial properties via various mechanisms, including (i) physically damaging bacterial cells, (ii) causing oxidative stress via the formation of reactive oxygen species, (iii) releasing free metal ions with direct antibacterial activity,^9^ and in the case of iron and iron oxide nanoparticles, and (iv) localized heating due to magnetic properties (magnetic hyperthermia).^10–12^ Moreover, spherical magnetic nanoparticles have recently been used to penetrate biofilms, thereby producing channels and improving antibiotic accessibility and bacterial eradication.^13–15^

By contrast, nonspherical (anisotropic) magnetic nanoparticles possess unique physical properties due to their shape. They can be prepared using a magnetic field-assisted sol–gel approach that enables good control over the length/diameter ratio.^16^ Additionally, superparamagnetic nanoparticles behave like nonmagnetic nanoparticles in the absence of the magnetic field, and thus enabling the easy preparation of colloidal suspensions, which can be exploited for the preparation of otherwise challenging ferromagnetic particles. The spatial orientation of anisotropic magnetic nanoparticles can be controlled remotely by applying a directed magnetic field that aligns the particles parallel to their long axis.^17^ Exposure of the anisotropic magnetic nanoparticles to a rotating magnetic field results in their rotation and generation of magnetic torque.^18^ This torque can produce forces that induce structural changes in soft matter, such as hydrogels and biofilms.^19^ In line with this, mechanical biofilm disruption was achieved using 70-nm ferromagnetic nanocrystals in a rotating magnetic field.^20^ Multifunctional nanoparticles with iron oxide cores and spiky polydopamine-gold coatings exhibited strong photothermal and magnetomechanical effects, achieving biofilm removal rates of over 50% in rotating magnetic fields.^21^ Nickel et al.^22^ used a rotating magnetic field to enhance nanoparticle movement through biofilms, maximizing physical damage. Anisotropic nanoparticles were the most effective in biofilm eradication, particularly when supported by the concomitant delivery of a biocide.

We have previously reported the synthesis of similar anisotropic superparamagnetic iron oxide nanoparticles (nanochains) that can rotate when exposed to a low-intensity (<20 mT) and low-frequency (<10 Hz) rotating magnetic field.^17^ These nanochains were used to mechanically damage planktonic *Staphylococcus epidermidis* biofilms and significantly improved the efficacy of methicillin (decreasing the viability by 99.99%). The aim of this study was to explore whether remotely magnetically guided nanochains’ movements can generate sufficient mechanical forces to effectively loosen, disrupt, and disperse soft matter such as attached biofilms. For that purpose, we systematically evaluated the efficacy of three nanochain types in mechanically removing prewashed attached biofilms of three bacterial species (*Escherichia coli*, *Lactococcus lactis*, and *Pseudomonas fragi*) under different conditions. In addition, the safety of these nanochains was evaluated using the Caco-2 cell line.

## Materials and Methods

### Preparation of Fluorescent Bacterial Strains

*Escherichia coli* DH5α was transformed with pSEUDO-CP25-GFP (a derivative of pSEUDO-GFP^23^ with the CP-25 promoter (CTTTGGCAGTTTATTCTTGACATGTAGTGAGGGGGCTGGTATAATCACATAGTACTGTT) inserted upstream of the *gfp* gene via the *Bam*HI/*Eco*RI sites) by heat shock. *Lactococcus lactis* NZ9000 was transformed with pNZ-ldh-mCherry^24^ by electroporation,^25^ using a BTX Gemini X^2^ Electroporation System (Bio-Rad, Hercules, CA, USA). *Pseudomonas fragi* ATCC 4973 was transformed with pBBR1MCS2-mCherry (a derivative of pBBR1MCS2 ^26^ with RBS (TAAGGAGGTTTTCTA)-mCherry gene fusion inserted via the *Kpn*I/XbaI sites).

### Bacterial Cultures

Bacteria were revitalized by streaking frozen bacterial cultures (−80 °C) on the following agar plates: lysogeny broth (LB) agar with 150 μg/mL erythromycin (for *E. coli* DH5α), LB agar with 100 μg/mL kanamycin (for *P. fragi* ATCC 4973), and M17 (Merck) agar with 0.5% glucose (GM17) and 10 μg/mL chloramphenicol (for *L. lactis* NZ9000). Plates were incubated for 16–18 h at 37 °C (*E. coli*) or 30 °C (*L. lactis* and *P. fragi*) and stored at 4 °C until further use (not more than 1 week). Single colonies from plates with revitalized bacteria were inoculated into 5 mL of either LB (for *E. coli* and *P. fragi*) or GM17 (for *L. lactis*) broth and supplemented with appropriate antibiotics (as specified above) and incubated overnight (16–18 h). *E. coli* was incubated at 37 °C with shaking at 190 rotations per minute (rpm), *P. fragi* was incubated at 30 °C with shaking at 130 rpm, and *L. lactis* was incubated at 30 °C without shaking.

### Biofilm Formation

To initiate biofilm formation, overnight cultures of each species (*E. coli*, *P. fragi*, and *L. lactis*) were diluted to a final optical density (OD) of 0.1 in growth media with appropriate antibiotics (LB with 150 μg/mL erythromycin for *E. coli*, LB with 100 μg/mL kanamycin, and GM17 with 10 μg/mL chloramphenicol for *L. lactis)*. These diluted cultures were pipetted into a black 96-well plate (Nunc) at 100 μL/well and incubated for 1 h at 37 °C (for *E. coli*) or at 30 °C (for *P. fragi* and *L. lactis*). After 1 h of adhesion, the media were replaced with 100 μL of fresh media, and the cultures were incubated for 24 h at 37 °C (for *E. coli*) or at 30 °C (for *P. fragi* and *L. lactis*) to form biofilms.

### Materials for Synthesis, Coating, and Functionalization of Magnetic Nanochains

Iron­(III) sulfate hydrate and iron­(II) sulfate heptahydrate were purchased from VWR Chemicals. Aqueous ammonia solution (25%) and dichloromethane (99.5%) were obtained from J.T. Baker. The following reagents were sourced from Sigma-Aldrich: tetraethyl orthosilicate (TEOS), *N*,*N*-diisopropylethylamine (≥99%), absolute ethanol, polyvinylpyrrolidone (PVP, *M*_w_ = 40 kDa), and triethanolamine (≥99%). Cetyltrimethylammonium bromide (CTAB, 98%) was supplied by Thermo Scientific, hydrobromic acid (48%) was obtained from Fluka, and tris­(hydroxymethyl)­aminomethane (99.8%) was obtained from Acros Organics. Cyclohexane (99.8%) was purchased from Carlo Erba Reagents. Hexadecyltrimethylammonium *p*-toluenesulfonate (99.0%) and *N*,*N*-dimethylformamide (DMF, 99.9%) were acquired from Merck. Ammonium nitrate (99+%) was obtained from Chemlab, and both (3-aminopropyl)­triethoxysilane (APTES, 98%) and succinic anhydride (99%) were purchased from Alfa Aesar.

### Magnetic Nanochain (NC) Synthesis

The synthesis of magnetic nanochains is based on a dynamic magnetic assembly process, where thin silica permanently connects and fixates the temporarily assembled nanoparticle clusters in a magnetic field. Since individual superparamagnetic maghemite nanoparticles (size ∼10 nm) are not sufficiently guidable by a magnetic field to be assembled into chain-like linear nanostructure, several nanoparticles (approximately 75 maghemite nanoparticles) are previously self-assembled into larger spherical nanoparticle clusters as the first step in the process, described in our previous publications in detail.^27,28^ Briefly, the nanoparticle clusters were synthesized through the self-assembly of primary maghemite (γ-Fe_2_O_3_) nanoparticles, followed by a thin silica coating. Initially, individual maghemite nanoparticles were prepared via coprecipitation from an aqueous solution. A solution containing Fe^2+^ (0.027 mol/L) and Fe^3+^ (0.023 mol/L) ions was precipitated using concentrated ammonia (25 wt %) in a two-step process. In the first step, the pH was raised to 3 and maintained for 30 min to allow the precipitation of iron hydroxides. In the second step, the pH was further increased to 11.6, promoting the oxidation of iron­(II) hydroxide by atmospheric oxygen, resulting in the formation of a spinel-phase product. After a 30-min aging period, the nanoparticles were thoroughly washed with a diluted ammonia solution (pH 10.5) and dispersed in distilled water (10 g/L). The nanoparticle clusters, with a mean size of ∼100 nm (>100 particles counted on TEM images), are further assembled into linear and rigid magnetic nanochains fixated by very thin (<5 nm) silica in a magnetic field, in accordance with an established method.^16^ To synthesize nanochains, the aqueous suspension of nanoparticle clusters was first transferred into a polyvinylpyrrolidone (PVP) solution at pH 4.3 while the resulting sol–gel mixture was stirred nonmagnetically at 250 rpm. Nanochain formation was performed at a PVP concentration of 1.25 × 10^4^ M, under a magnetic field of (5.2 ± 1.2) × 10^4^ A/m for 85 min following the addition of tetraethyl orthosilicate (TEOS). The nanoparticle cluster concentration was 1.6 × 10^– 8^ M, and the TEOS concentration was 60 mM. TEOS was added 10 min after the nanoparticle clusters were transferred into the PVP solution. After 80 min of TEOS exposure, the pH was adjusted to 8.5 using a 0.5% ammonia solution. After the reaction, the magnetic nanochains (NC) were washed and resuspended in distilled water to a final concentration of 10 g/L. These nanochains are labeled as NC.

### Magnetic Nanochain Coating with Functional Silica

Functional silica coatings with varying roughness were deposited on the surface of as-synthesized magnetic nanochains with a very thin primary fixating silica layer. The coating procedures were based on our previously published protocols, with minor modifications as outlined in the subsequent methodological descriptions.^29^

#### Nanochains with Smooth Silica Coating Functionalized with COOH Groups (NC-Si)

For the smooth silica coating, 100 mg of nanochains (NC) were resuspended in distilled water to a final volume of 114 mL. The suspension was sonicated for 1 min, after which 18 mL of aqueous ammonia solution (25 wt %) was added. Following another 1 min of sonication, 300 mL of 0.02 M TEOS in ethanol was introduced into the mixture. The reaction flask was left under continuous stirring overnight. The following day, the particles were washed twice with ethanol, followed by two washes with distilled water. Finally, the silica-coated nanochains were resuspended in water to a final concentration of 10 mg/mL. These nanochains were then functionalized with APTES to introduce amine groups that subsequently reacted with succinic anhydride in order to get free surface carboxyl groups on the nanochains. These functionalization procedures have been slightly modified according to our published protocols.^30,31^ Briefly, 100 mg of nanochains were dispersed in a mixture of 25 mL ethanol and 25 mL distilled water. The suspension was placed in an oil bath maintained at 50 °C. Then, 0.9 mL of aqueous ammonia solution (25 wt %) was added, followed by 0.3 mL of APTES. The reaction mixture was stirred overnight at 50 °C. The next day, the particles were washed twice with distilled water and resuspended in 20 mL of DMF. To this suspension, 25 mL of additional DMF and 0.3 mL of *N*,*N*-diisopropylethylamine were added. The flask was placed in an oil bath at 50 °C, and 200 mg of succinic anhydride, dissolved in 5 mL of DMF, was introduced. The reaction mixture was stirred at 50 °C for 3 h. After the reaction, the nanochains were washed and resuspended in distilled water to a final concentration of 10 mg/mL. These magnetic nanochains are labeled as NC-Si.

#### Nanochains with Moderately Rough Silica Coating with COOH Groups (NC-ToSi)

For a moderately rough silica coating on nanochains (NC), a 52 mL of a 0.025 M hexadecyltrimethylammonium *p*-toluenesulfonate solution was first prepared and placed in an oil bath at 50 °C. Then, 100 mg of nanochains were added to the previously prepared hexadecyltrimethylammonium *p*-toluenesulfonate solution, followed by an addition of TEOS (2.5 mL), and after 10 min, a triethanolamine base (6.25 μL) was added. The reaction was left stirring overnight at 50 °C. The next day, the nanochains were washed twice with distilled water and resuspended to a final concentration of 10 mg/mL. In order to remove residual templating surfactants, an additional washing step with ammonium nitrate is required. Briefly, 2 g of ammonium nitrate was dissolved in 100 mL of ethanol. The mixture was heated to 60 °C until the chemical was fully dissolved. Then, 100 mg of the nanochains were added to the solution, and the suspension was stirred at 60 °C for 1 h. Following this treatment, the nanochains were washed twice with ethanol and resuspended in 50 mL of ethanol. If the isoelectric point of the washed nanochains is at pH >4.0, the process is repeated. After the final washing step, the particles were resuspended in distilled water to a final concentration of 10 mg/mL. The introduction of COOH groups followed exactly the same protocol as that described for NC-Si synthesis. After COOH functionalization, these nanochains are labeled as NC-ToSi.

#### Nanochains with Highly Rough Silica Coating Functionalized with COOH Groups (NC-Hex)

For highly rough silica coating on nanochains (NC), 75 mL of a 0.26 M CTAB solution was first prepared. Second, 137 mg of tris­(hydroxymethyl)­aminomethane (TRIS) was weighed into a small vial and dissolved in 5 mL of the previously prepared CTAB solution. Then, 100 mg of nanochains were added to the first prepared CTAB solution, followed by the gradual addition of the diluted TRIS-CTAB solution. The resulting suspension was sonicated for 1 min. The mixture was transferred to a round-bottom flask equipped with a condenser, placed in an oil bath at 50 °C, and stirred at 600 rpm for 10 min. A solution of 0.938 mL TEOS in 11 mL of cyclohexane was then added to this suspension of nanochains. The reaction was allowed to proceed with continuous stirring for 1.5 h, after which a second identical amount of TEOS in cyclohexane was added. The reaction mixture was left stirring overnight at 50 °C. The following day, the particles were washed twice with distilled water and resuspended to a final concentration of 10 mg/mL. In order to remove residual templating surfactants, an additional washing step with ammonium nitrate is required. Briefly, 2 g of ammonium nitrate was dissolved in 100 mL of ethanol. The mixture was heated to 60 °C until the chemical was fully dissolved. Then, 100 mg of the nanochains were added to the solution, and the suspension was stirred at 60 °C for 1 h. Following this treatment, the nanochains were washed twice with ethanol and resuspended in 50 mL of ethanol. In case the isoelectric point of washed nanochains is at pH >4.0, the process is repeated. After the final washing step, the particles were resuspended in distilled water to a final concentration of 10 mg/mL. The introduction of COOH groups followed exactly the same protocol as described for NC-Si synthesis. After COOH functionalization, these nanochains are labeled as NC-Hex.

### Transmission Electron Microscopy

Transmission electron microscopy (TEM) analyses were obtained using a transmission electron microscope (JEM, Jeol 2100) coupled with energy-dispersive X-ray spectroscopy (EDXS, JED 2300 EDS). For all samples, TEM grids were prepared by depositing a few drops of the diluted suspensions onto carbon-coated copper grids, followed by air-drying at room temperature.

### Scanning Electron Microscopy

Scanning electron microscopy (SEM) images were acquired using a Thermo Fisher Verios 4G HP scanning electron microscope. For SEM analysis, the samples and grids were prepared following the same procedure used for TEM analysis.

### Zeta Potential Measurements

Zeta potential measurements were obtained on a Litesizer 500 (Anton Paar). The nanochains were diluted to a concentration of 0.025 mg/mL in a 10 mM KCl solution and transferred to a beaker for automated titration analysis.

### Vibrating Sample Magnetometry (VSM) Measurements

Magnetic properties of the samples were characterized by using a LakeShore Series 7400 Vibrating Sample Magnetometer. A known mass of dried sample (10–20 mg) was tightly packed into a sample holder and placed within the VSM setup. Measurements were conducted in continuous loop mode over a magnetic field range of −10 to +10 kOe.

### Inductively Coupled Plasma Atomic Emission Spectrometry (ICP-AES) Measurements

The iron concentrations in the samples were determined by elemental analysis using inductively coupled plasma atomic emission spectrometry (ICP-AES) (iCAP6200 duo, Thermo Fisher Scientific, Waltham, MA, USA). Samples were dispersed in a HNO_3_ and HCl solution (5 mL), the acid solution was evaporated, and 5 mL of a 1% HCl solution was added for the analysis.

### Functionalized Nanochain Preparation for Biofilm Assay

All three types of functionalized nanochains (NC-Si, NC-ToSi, and NC-Hex) were initially suspended in ethanol to avoid microbial contamination and sonicated for 3 min at 37 kHz and 30% power (Elmasonic P30 H, Elma) before use. After vortexing, they were transferred to microcentrifuge tubes on a magnetic stand, and an equal volume of PBS was added. Once the suspension cleared , the supernatant was carefully removed, and PBS was added at twice the initial volume to wash away residual ethanol. The PBS was then removed, and the nanochains were adjusted to a concentration of 10 mg/mL with fresh PBS.

### Treatment of Biofilm with Functionalized Nanochains and Fluorescence Measurements

After 24 h of biofilm formation on microtiter plates (see the section Biofilm Formation), the wells were washed once with 100 μL of PBS, followed by the addition of 90 μL of PBS. Nanochain suspensions (10 μL with a final concentration of 1 mg/mL) or PBS (control) were added to each well. The plates were then placed on either of the two magnetic stirrers: a 96-well stirrer (MIXdrive 96 MTP, 2mag AG at 100% power) and a classic lab magnetic stirrer (Rotamix S-10, Domel). Two stirring speeds (300 and 600 rpm) and two durations of treatment (30 and 60 min) were used, resulting in four different conditions for each stirrer. After treatment on the stirrers, the contents of the wells containing detached bacteria were collected and transferred to a new black 96-well microtiter plate (Nunc). Here, the fluorescence was measured using an M-1000 microplate reader (Tecan) with excitation and emission wavelengths set at 488/509 nm for GFP and 587/610 nm for mCherry with 9- reads per-well setting, and bacteria were quantified by determining colony-forming units (CFUs; see the section CFU Counting). Fresh PBS (100 μL) was added to the empty wells of the original microtiter plate containing the remaining biofilm, and fluorescence was measured as described above. Afterward, the biofilm was dissociated and homogenized using sonication by placing the plates in an ultrasonic bath for 30 min (37 kHz, 30% power). To protect samples from contamination, plates were sealed with an air-permeable membrane and wrapped in parafilm. Dissociated bacteria were quantified by CFU counting.

### CFU Counting

After sonication, serial 10-fold dilutions (ranging from 10^–1^ to 10^–9^) of each bacterial suspension were prepared in sterile 96-deep well plates by mixing 50 μL of samples with 450 μL of PBS. Dilutions were then transferred to agar plates in 10 μL aliquots using a multichannel pipette, with all eight dilutions of each sample pipetted simultaneously to ensure consistency. *E. coli* and *P. fragi* were plated on LB agar plates, whereas *L. lactis* was plated onto GM17 agar plates. Plates were dried in a laminar flow hood before incubation at 37 °C for *E. coli* and 30 °C for *P. fragi* and *L. lactis* for 16–18 h. Colonies were counted at dilutions within the range of 1–30 CFUs/spot to ensure accuracy and reproducibility within the quantifiable range.

### Caco-2 Cell Line, Culturing, and Preparation of Monolayer

Human colon adenocarcinoma Caco-2 cells (HTB-37; ATCC) were cultured in Dulbecco’s modified Eagle’s medium (DMEM) with high glucose and GlutaMAX (Gibco) supplemented with 20% (v/v) fetal bovine serum (Gibco), 100 U/mL penicillin, and 100 μg/mL streptomycin (Gibco). The cells were seeded in 96-well transparent plates at a density of 1 × 10^5^ cells/mL in a volume of 100 μL/well. The plates were incubated at 37 °C in a humidified atmosphere containing 5% CO_2_ for 21 days to promote the monolayer formation. The culture medium was replaced every 2 days.

### Incubation of the Caco-2 Cell Monolayer with Nanochains

Nanochains (NC-Si, NC-ToSi, and NC-Hex) were suspended in ethanol, sonicated for 3 min, and washed with PBS as described above. After the removal of PBS, nanochains were resuspended in fresh DMEM to a final concentration of 1 mg/mL. To test cytotoxicity, six serial 2-fold dilutions of the initial nanochain suspension were prepared in DMEM, yielding concentrations of 0.500, 0.250, 0.125, 0.063, 0.031, and 0.016 mg/mL. To ensure homogeneity, nanochain suspensions were thoroughly vortexed before the addition to the cells. Caco-2 cell monolayers were exposed to dilutions of nanochains by replacing their culture medium with fresh DMEM containing nanochains. Cells incubated in DMEM without nanochains served as a negative control, whereas the toxic polypeptide melittin from bee venom (10 and 20 μg/mL, Merck) was used as a positive control. The plates were exposed to magnetic stirring at 300 rpm for 1 h using a magnetic stirrer at 100% power (MIXdrive 96 MTP, 2mag AG) at room temperature, followed by a further 23 h of incubation at 37 °C in a CO_2_ incubator (5% CO_2_; CB-S 170, Binder). Alternatively, the plates were incubated for 24 h at 37 °C in a CO_2_ incubator (5% CO_2_; CB-S 170, Binder) without stirring. After incubation, the cell viability of Caco-2 cells was evaluated using the resazurin assay.

### Resazurin Assay

Resazurin (Merck) was dissolved in DPBS at a concentration of 0.4 mg/mL. Aliquots (10 μL) of the resazurin solution were added to each well containing a Caco-2 monolayer and incubated for 2 h at 37 °C in a CO_2_ incubator. After incubation, the supernatants were transferred to black microtiter plates (Nunc). Fluorescence measurements (excitation: 550 nm, emission: 590 nm) were conducted using a microplate reader (M-1000, Tecan).

### Light Microscopy

The integrity of the Caco-2 cell monolayer after exposure to nanochains with or without stirring was inspected under a light microscope (CKX53 equipped with DP23 digital camera, Olympus), and structural damage was assessed. Melittin (10 μg/mL) was included as a positive control. Images were captured at 4×, 10×, and 20× magnifications.

### Statistical Analyses

Statistical analyses were performed using the GraphPad Prism 6. Data are presented as mean ± standard error of the mean from three biological and three technical repeats. One-way ANOVA with Dunnett’s posthoc test was used to determine the significance of the differences between the treated samples and their respective controls. Differences were shown using a compact letter display; groups that do not share a letter are significantly different (*p* < 0.05). Alternatively, significant difference is depicted by asterisks (***p < 0.001; ****p < 0.0001).

## Results

### Synthesis and Functionalization of Superparamagnetic Nanochains

Three different types of functionalized nanochains were prepared in our study, and their magnetomechanical actuation effects on bacterial cells and biofilm elimination efficacy were investigated in detail. Primary nanochains were prepared by the magnetic assembly of several nanoparticle clusters (usually 4–6) that were simultaneously aligned in a chain-like formation and fixed with ultrathin silica to form a permanently stable nanostructure (Figure 1A). In the first step of the synthesis, iron oxide nanoparticles were produced and self-assembled into spherical nanoparticle clusters with an average size of approximately 100 nm (Figure 1B). The resulting primary nanochains, therefore, have a typical length ranging from 0.5 to 1 μm. These primary nanochains were then coated with silica shells exhibiting smooth, moderately rough, and highly rough morphologies, and were named NC-Si, NC-ToSi, and NC-Hex, respectively (Figure 2). These nanochains were subsequently functionalized with surface carboxyl groups, which ensured good colloidal stability in different culture media. The elongated (anisotropic shape) structure of nanochains and their high magnetic responsiveness allowed them to align and rotate when actuated by a rotating magnetic field, thereby generating mechanical forces sufficient for planktonic biofilm dispersal.^17^

**Figure 1:**
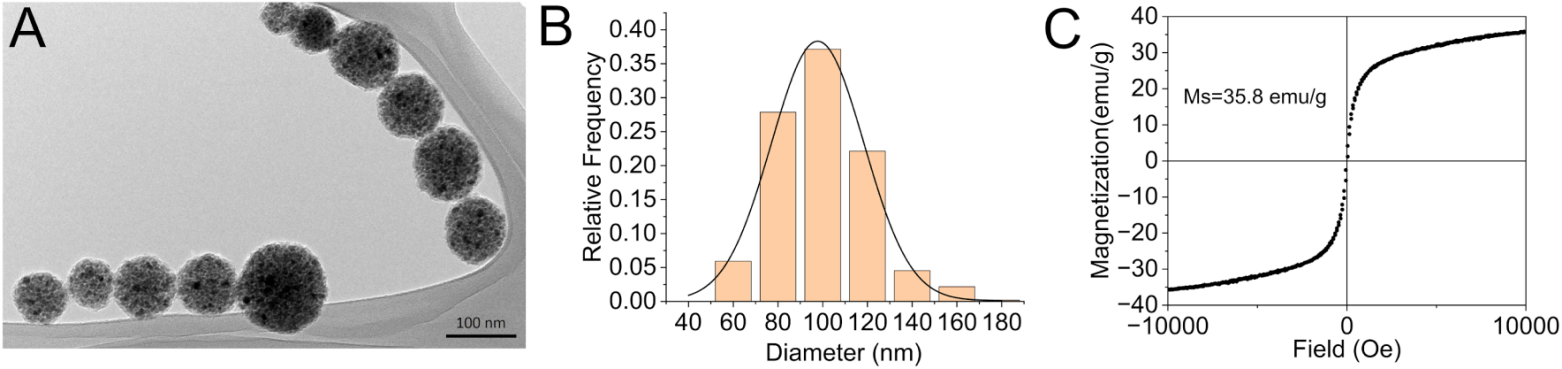
TEM image of the as-synthesized nanochains (A), size distribution of iron oxide nanoparticle clusters (from which nanochains are synthesized) (B), and room-temperature measurements of the mass magnetization as a function of the magnetic field for the as-synthesized nanochains (C).

**Figure 2:**
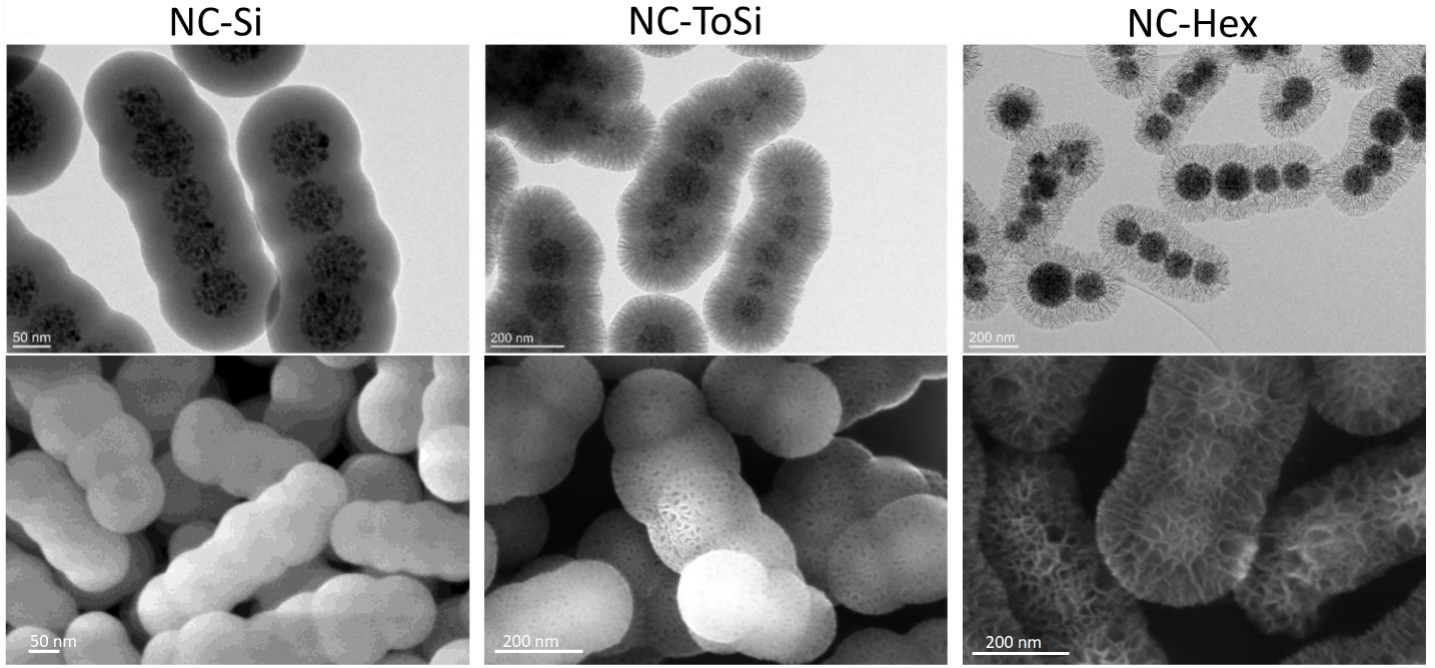
Transmission electron microscopy (TEM; top) and scanning electron microscopy (SEM; bottom) images of the functionalized nanochains: smooth NC-Si, moderately rough NC-ToSi, and highly rough NC-Hex.

All three types of functionalized nanochains were fully characterized. The vibrating sample magnetometry (VSM) measurement of primary nanochains (Figure 1C) shows no hysteresis loop, which means the nanochains exhibit superparamagnetic behavior. The absence of remanent magnetization confirms that the nanochains do not retain net magnetic moment once the external magnetic field is removed. Such behavior is crucial for biomedical applications because it prevents the nanochains from aggregating due to attractive magnetic dipole interactions, ensuring they remain colloidally stable and well-dispersed in physiological environments. These primary nanochains have a saturation magnetization value of 35.8 emu/g and relatively large volume, reflecting their quick magnetic response to a magnetic field. Taking into account the magnetic properties of randomly oriented nanochains, magnetically aligned nanochains, and their building blocks, it can be assumed that nanochains readily and efficiently respond to dynamic changes in the direction of a rotating magnetic field, even at relatively low rotation speeds (Section S1). This dynamic magnetic responsiveness influences the magnetorheological behavior of the nanochains and facilitates effective transmission of magnetic energy into mechanical torque, which plays a key role in the physical disruption of soft matter, such as bacterial biofilms.

To investigate the effects of nanochains’ surface roughness on biofilm dispersal efficacy, nanochains were coated with functional silica exhibiting varying surface morphologies, ranging from very smooth to highly rough, as clearly seen in the electron microscopy images in Figure 2. The average final silica shell thicknesses were 45, 106, and 75 nm for NC-Si, NC-ToSi, and NC-Hex, respectively.

Following coating with functional silica, the saturation magnetization values decreased to 13.1, 7.4, and 15.0 emu/g for NC-Si, NC-ToSi, and NC-Hex, respectively (Figures 3A and S1 for mass-normalized magnetization). For all subsequent experiments, the initial mass concentration of NC-Si, NC-ToSi, and NC-Hex was set to 1.0 mg/mL, corresponding to Fe concentrations of 2.524 mmol/L, 1.426 mmol/L, and 2.890 mmol/L, respectively. This mass magnetization reduction is expected, as the addition of nonmagnetic silica increases the total mass of the particles without contributing to their magnetization. The largest magnetization decrease was observed for NC-ToSi, which correlates well with its significantly thicker silica shell. In contrast, the NC-Hex exhibited the highest magnetization among the coated samples, although the silica layer in this sample is also relatively thick. This could be explained by highly rough morphology of the silica shell of NC-ToSi and NC-Hex (average pore sizes 9.6 ± 2.1 nm and 16.6 ± 5.3 nm, respectively; Figure 3B,C) meaning that the silica is less dense due to empty compartments, as clearly seen in SEM images in Figure 2. Importantly, all functionalized nanochains retained their superparamagnetic behavior, as evidenced by the absence of hysteresis in the vibrating sample magnetometry measurements.

**Figure 3:**
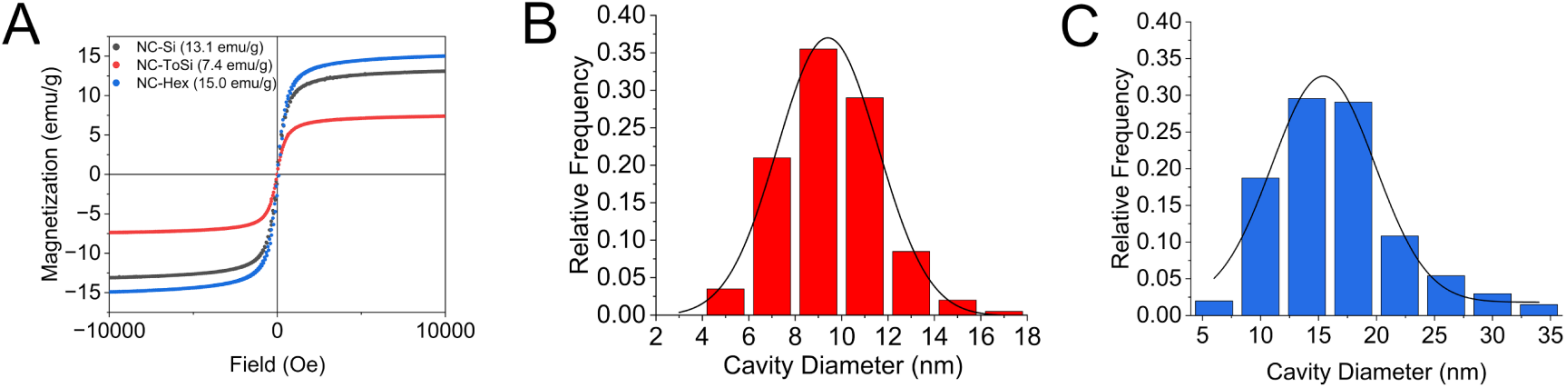
Room-temperature measurements of the mass magnetization as a function of magnetic field for all three types of functionalized nanochains (NC-Si, NC-ToSi, and NC-Hex; A), and cavity size distribution for NC-ToSi (B) and NC-Hex (C).

Apart from improving colloidal stability in complex culture media and providing mechanical stability to the nanostructure, the silica shell also enables a versatile platform for further surface functionalization. Thus, the nanochains with functional silica were further functionalized with APTES, followed by a reaction with succinic anhydride (SA), which introduced carboxylic groups onto the nanochain surface. This surface modification increased the absolute values of zeta potential, which could enhance repulsive electrostatic interactions between the nanochains and negatively charged bacterial membranes and minimize nonspecific binding during biofilm dispersal.

The surface functionalization of the nanochains was confirmed indirectly by measuring the zeta potential after every reaction step. Although it is known that zeta potential analysis is primarily designed for spherical nanoparticle characterization, the technique still provides clear and consistent trends for our anisotropic nanochains, confirming the successful surface functionalization. The three types of nanochains exhibit a shift in isoelectric points (IEPs) after functionalization. Silica-coated nanochains exhibited low IEP values (pH between 3.0 and 3.7) (Figure S2), consistent with the negatively charged character of the silica surface in the physiological pH range. Upon APTES functionalization, the IEP shifted to a higher pH between 10.3 and 10.6, indicating the successful introduction of amine groups, as seen in Figure S3. Following succinic anhydride functionalization, the IEP decreased to a lower pH again, reflecting the presence of carboxyl groups on the surface, as seen in Figure 4. These trends confirmed that each step of the surface functionalization was successful.

**Figure 4:**
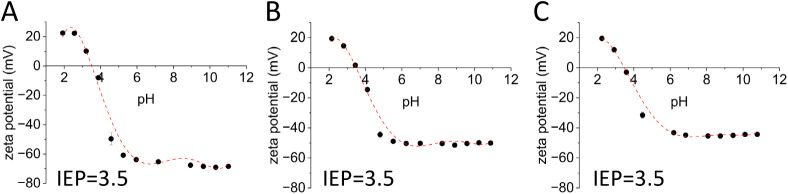
Zeta potential measurements of functionalized nanochains NC-Si (A), NC-ToSi (B), and NC-Hex (C).

### Experimental Design for Magnetomechanical Removal of Bacterial Biofilms with Functionalized Nanochains

The efficacy of functionalized nanochains in the mechanical removal of bacterial biofilms was assessed in a rotating external magnetic field by systematically testing a set of parameters (Figure 5). Functionalized nanochains NC-Si, NC-ToSi, and NC-Hex with varying roughness of the outer silica layer were included. Two different rotating magnetic fields were generated using either a 96-well magnetic stirrer (with a weaker magnetic field, i.e., with a mean of 2.2 mT/well and providing rotation of nanochains in each well of the microtiter plate) or a classic lab magnetic stirrer (with a stronger magnetic field, i.e., with a mean of 47.0 mT/well, relatively large magnetic field gradient of 0.5 T/m (see Section S3) and providing sweeping motion of nanochains in the central part of the microtiter plate). Details of the magnetic field strength are shown in Figure S4. Biofilms were exposed to the rotating magnetic field at two different speeds (300 rpm, 600 rpm) and two different durations (30 min, 60 min). Three different model nonpathogenic bacterial species were included in the study: a Gram-negative laboratory strain of intestinal commensal *E. coli*, a Gram-negative strain of dairy spoilage-causing *P. fragi*, and a Gram-positive laboratory strain of milk-fermenting *L. lactis*. Biofilm removal was evaluated using two methods: CFU counting and fluorescence measurement. For the latter, the bacteria were engineered to express Green Fluorescent Protein (GFP) or Red Fluorescent Protein (mCherry). Both methods were used to quantify the biofilm on the microplate surface and the number of detached bacterial cells in the supernatant.

**Figure 5:**
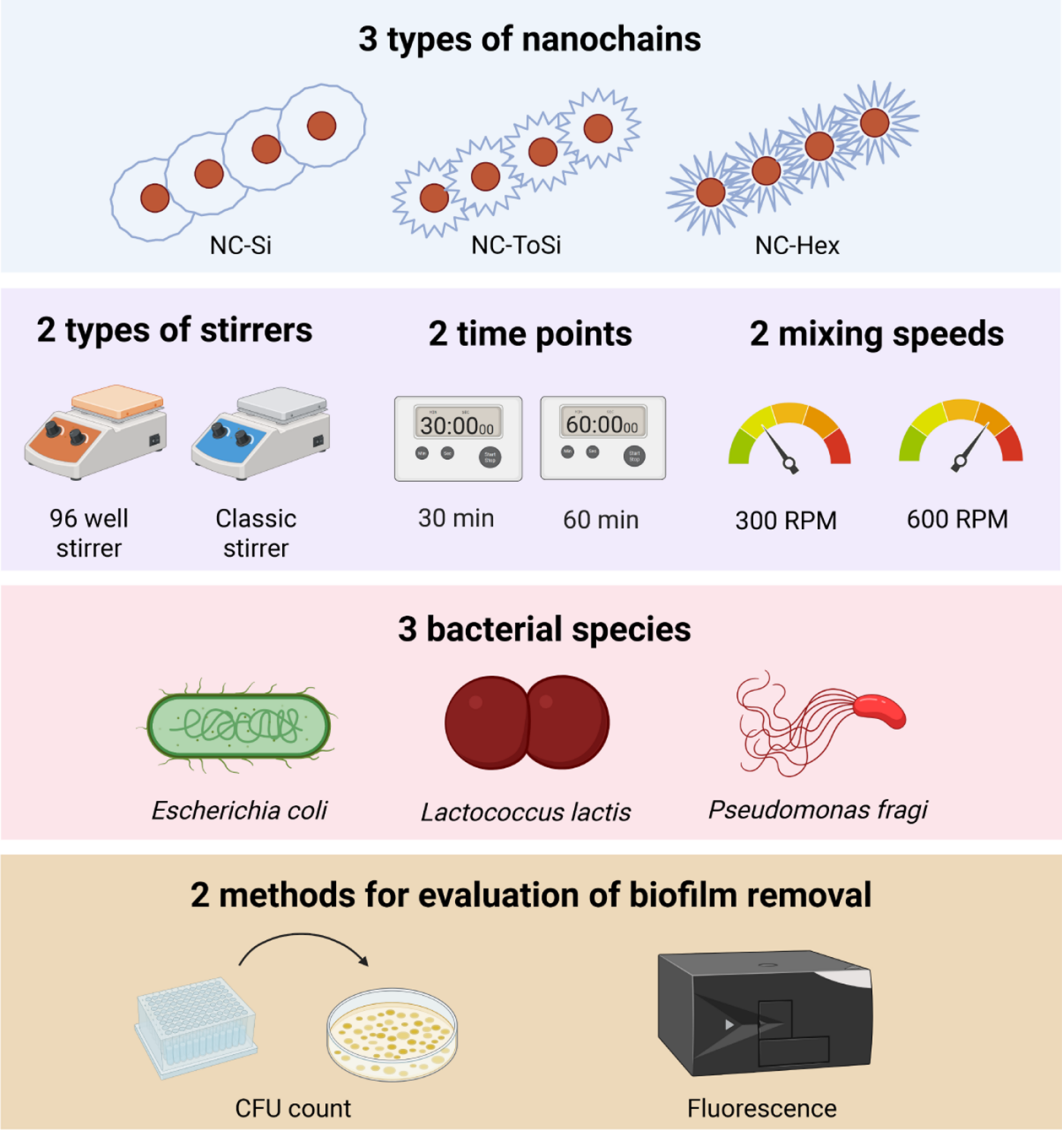
Schematic representation of the parameters tested in magnetomechanical removal of biofilms using functionalized magnetic nanochains.

### Quantification of Bacteria via Fluorescence Measurement

The expression of GFP in *E. coli* and mCherry in *L. lactis* and *P. fragi* was confirmed by measuring the fluorescence of various concentrations of bacteria. Linear increases in fluorescence intensities were observed as a function of bacterial concentration in the range of OD_600_ = 0.1–1.0 (Figure 6), indicating that fluorescence can be used to estimate the bacterial concentration. The fluorescence of mCherry in *P. fragi* was lower than the fluorescence in the other two species, possibly due to a lower expression level. This resulted in a larger standard deviation and a lower coefficient of determination in *P. fragi*.

**Figure 6:**
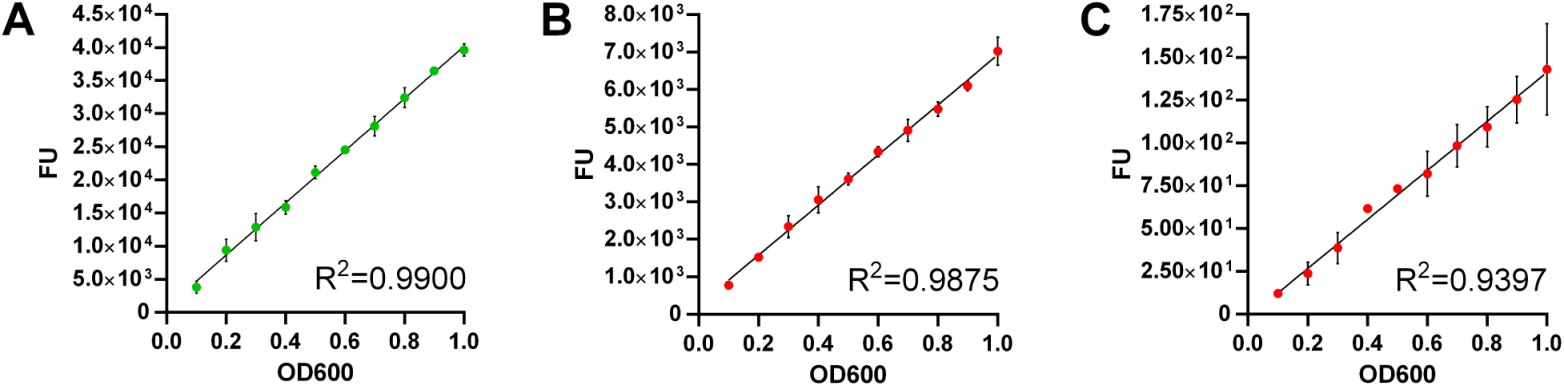
Fluorescence intensity (FU) of engineered bacteria expressing fluorescent proteins as a function of the bacterial concentration. (A) *E. coli* expressing GFP; (B) *L. lactis* expressing mCherry; (C): *P. fragi* expressing mCherry. *R* ^2^, coefficient of determination.

### Removal of *E. coli* Biofilms by Magnetomechanically Actuated Nanochains

The highest removal efficacy of *E. coli* biofilm (>90%) was achieved with the classic lab magnetic stirrer at 60 min and 600 rpm with significant reductions in biofilm observed through both CFU counts and fluorescence measurements (Figure 7). This suggests greater susceptibility of *E. coli* biofilms to disruption by sweeping motions in stronger magnetic fields at high speeds and longer durations. The 96-well stirrer was less effective, achieving removal rates of ∼80% at 300 rpm after 30 min with NC-ToSi and NC-Hex nanochains but not NC-Si, which showed no significant CFU decreases under the same conditions. Nanochain surface roughness might play a role under these conditions as a nanoscale feature that is important for improved mechanical biofilm loosening. Under these conditions, the results of the CFU and fluorescence measurements differed considerably. With longer treatment using the 96-well stirrer (300 rpm for 60 min), the removal efficacy was lower (50–80%), with good CFU–fluorescence match across all nanochain types. Higher stirring speed (600 rpm) with the 96-well stirrer showed diminished biofilm removal efficacy (∼50% or less) across all tested nanochains, suggesting that *E. coli* biofilms respond better to lower stirring speed. Supernatant analysis confirmed biofilm removal under most conditions. CFU counting showed a significant increase in the number of bacteria in the supernatants, confirming effective detachment of biofilm, particularly with the classic stirrer. The results obtained by measuring *E. coli* fluorescence in the supernatants mostly aligned with the CFU counts, highlighting the consistency of biofilm removal across methods. Overall, the data underscore the considerable susceptibility of *E. coli* biofilms to mechanical disruption and demonstrate that the classic stirrer at 60 min and 600 rpm emerged as the most effective system for *E. coli* biofilm removal.

**Figure 7:**
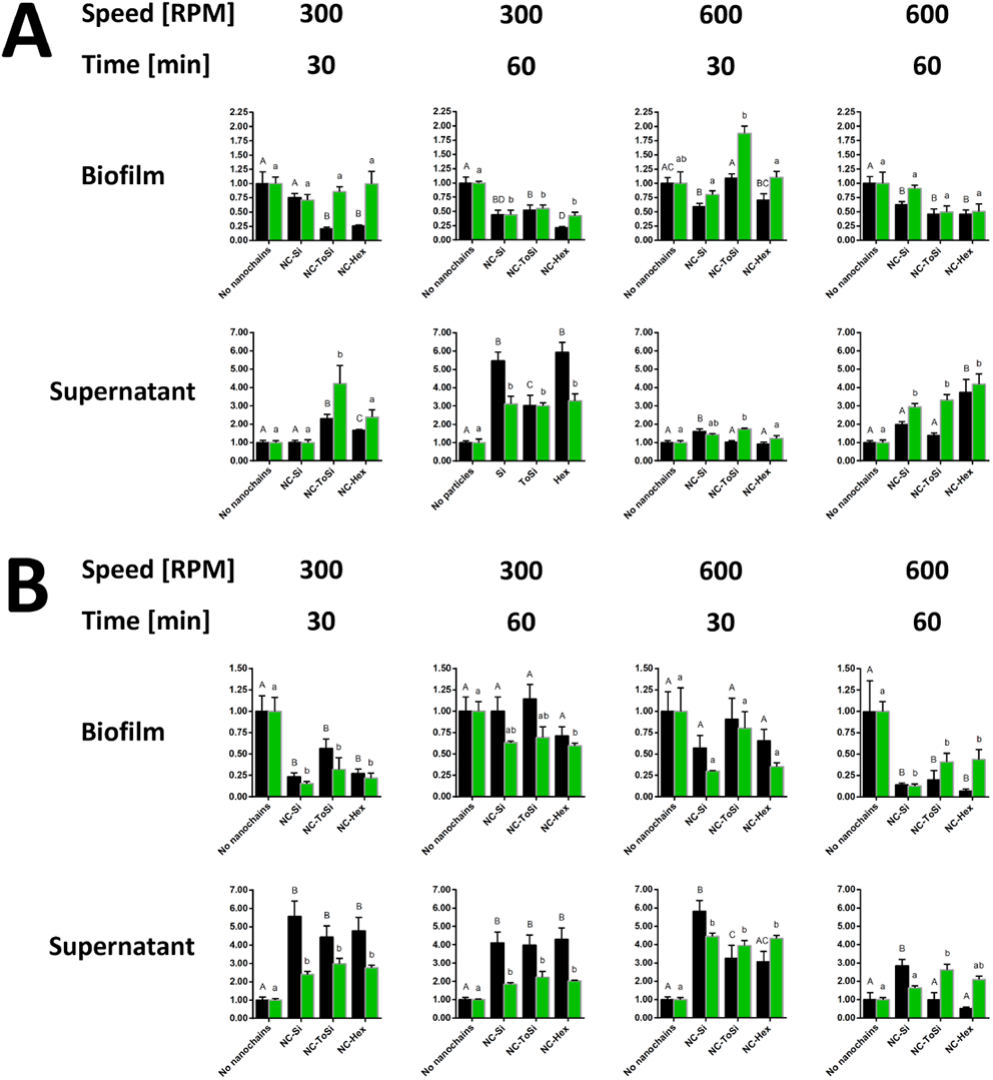
Treatment of *E. coli* biofilms with functionalized nanochains (NC-Si, NC-ToSi, and NC-Hex). A 96-well stirrer (A) or classic lab magnetic stirrer (B) was used. Bacteria were quantified by CFU counting (black bars) and fluorescence measurement (FU, green bars) in both biofilms and supernatants after treatment. Data from CFU counting and fluorescence were normalized relative to the respective values obtained in biofilm samples to which no particles were added. The significance of the differences was determined with one-way ANOVA with Dunnett’s posthoc test. Differences were shown using a compact letter display; groups that do not share a letter are significantly different (*p* < 0.05).

### Removal of *L. lactis* Biofilms by Magnetomechanically Actuated Nanochains

Maximum removal of *L. lactis* biofilms (<90%) was lower than that achieved in *E. coli*. The highest removal rates (75–85%) were observed using the 96-well stirrer for 60 min (both at 300 and 600 rpm) (Figure 8). Under these conditions, there were no significant differences between the three nanochain types, and the CFU and fluorescence results corresponded well. With the shorter treatment time (30 min), the removal efficacy with the 96-well stirrer decreased to 50–65% and was mostly not significant. Interestingly, after 30 min, NC-ToSi was the least effective. The treatment with the classic lab magnetic stirrer was less effective than with the 96-well stirrer. It was slightly more effective at the lower stirring speed (300 rpm; >50% removal) than at the higher stirring speed (600 rpm; <50% removal). Unlike the CFU results, the fluorescence results demonstrated no significant biofilm removal with the classic stirrer. The supernatant analysis after treatment with the 96-well stirrer mostly failed to mirror the biofilm removal observed by monitoring the remaining biofilm in the wells, particularly when using fluorescence. However, both the fluorescence and CFU results demonstrated that treatment with the classic stirrer resulted in significant increases in bacteria in the supernatant under three of the four conditions tested. Overall, despite the discrepancy between the results of the biofilms and the supernatants, *L. lactis* biofilms seem to be more susceptible to stirring motion (96-well stirrer) than *E. coli* biofilms, particularly with the longer treatment time (60 min).

**Figure 8:**
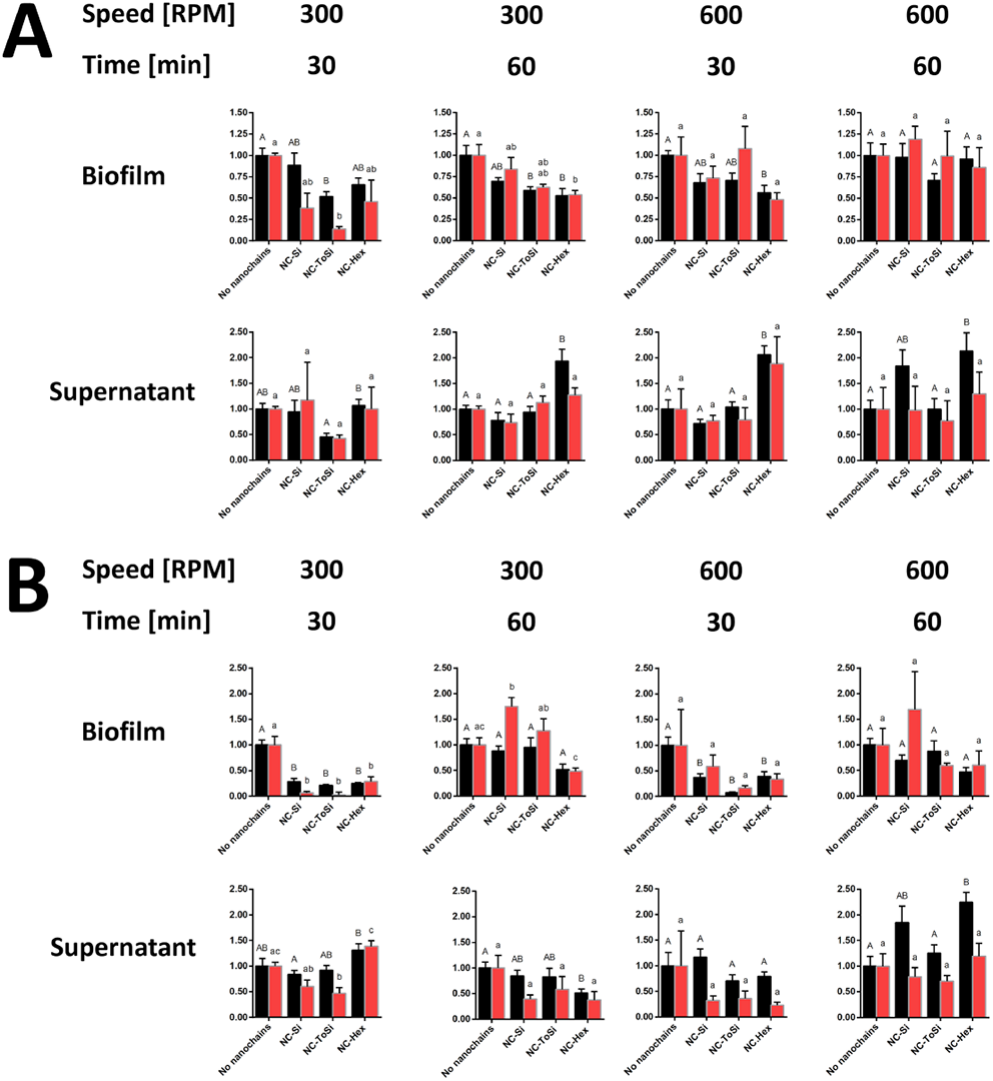
Treatment of *L. lactis* biofilms with functionalized nanochains (NC-Si, NC-ToSi, and NC-Hex). A 96-well stirrer (A) or classic lab magnetic stirrer (B) was used. Bacteria were quantified by CFU counting (black bars) and fluorescence measurement (FU, red bars) in both biofilms and supernatants after treatment. Data from CFU counting and fluorescence were normalized relative to the respective values obtained in biofilm samples to which no particles were added. The significance of the differences was determined with one-way ANOVA with Dunnett’s posthoc test. Differences were shown using a compact letter display; groups that do not share a letter are significantly different (*p* < 0.05).

### Removal of *P. fragi* Biofilms by Magnetomechanically Actuated Nanochains

The highest removal efficacy of *P. fragi* biofilms was similar to that achieved with *E. coli* (>90%). As with *E. coli*, the highest biofilm removal was achieved using the classic lab magnetic stirrer. Surprisingly, the shorter treatment duration (30 min) was more effective (70%–80% removal at 300 rpm and 60%–90% removal at 600 rpm) than the longer treatment (60 min), which resulted in mostly nonsignificant removal (Figure 9). When using the 96-well stirrer, a significant decrease in biofilm was observed in three of the four conditions tested; however, the removal rates were lower (30%–50%). The match between the CFU and fluorescence results was lower, and the decreased fluorescence was mostly not significant due to the higher variability. Discrepancies between the results of supernatants and direct biofilm assessment were observed, particularly when using a classic lab magnetic stirrer. Overall, *P. fragi* biofilms appear to be more similar to *E. coli* biofilms than to *L. lactis* biofilms regarding magnetomechanical removal. The classic stirrer was more effective; however, lower speeds and shorter treatment times were more suitable for the *P. fragi* biofilm removal. Nevertheless, substantial variability has to be considered when interpreting the results. In general, effective removal of biofilm was achieved with all three types of nanochains, but the effective conditions of removal differed among individual bacterial species.

**Figure 9:**
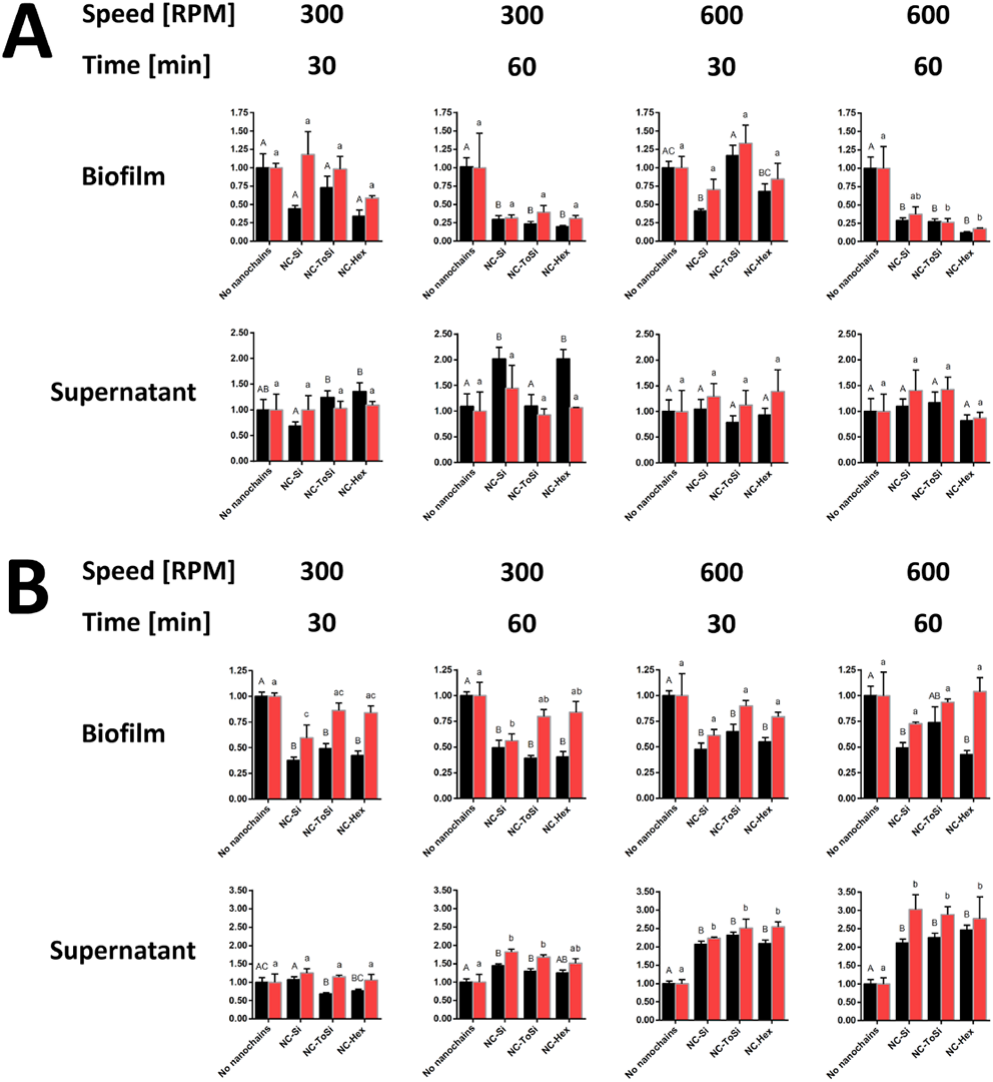
Treatment of *P. fragi* biofilms with functionalized nanochains (NC-Si, NC-ToSi, and NC-Hex). A 96-well stirrer (A) or classic lab magnetic stirrer (B) was used. Bacteria were quantified by CFU counting (black bars) and fluorescence measurement (FU, red bars) in both biofilms and supernatants after treatment. Data from CFU counting and fluorescence were normalized relative to the respective values obtained in biofilm samples to which no particles were added. The significance of the differences was determined with one-way ANOVA with Dunnett’s posthoc test. Differences were shown using a compact letter display; groups that do not share a letter are significantly different (*p* < 0.05).

### Functionalized Nanochains Do Not Affect Caco-2 Cell Viability or Monolayer Integrity

The safety of functionalized nanochains was confirmed in Caco-2 epithelial cells over a broad range of concentrations. Cell viability assay, performed by measuring the cell metabolic activity via resazurin reduction, revealed no statistically significant differences between treated and control cells, suggesting that the nanochains did not compromise cell viability after 24 h of incubation (Figure 10). Cell viability was retained regardless of magnetic stirring. Conversely, the cytotoxic peptide melittin decreased cell viability, confirming the validity of the assay. Nanochain dilutions without Caco-2 cells (cell-free blanks) were also tested and showed no fluorescence after incubation with resazurin, thus demonstrating that there is no interference of nanochains with fluorescence measurements (data not shown).

**Figure 10:**
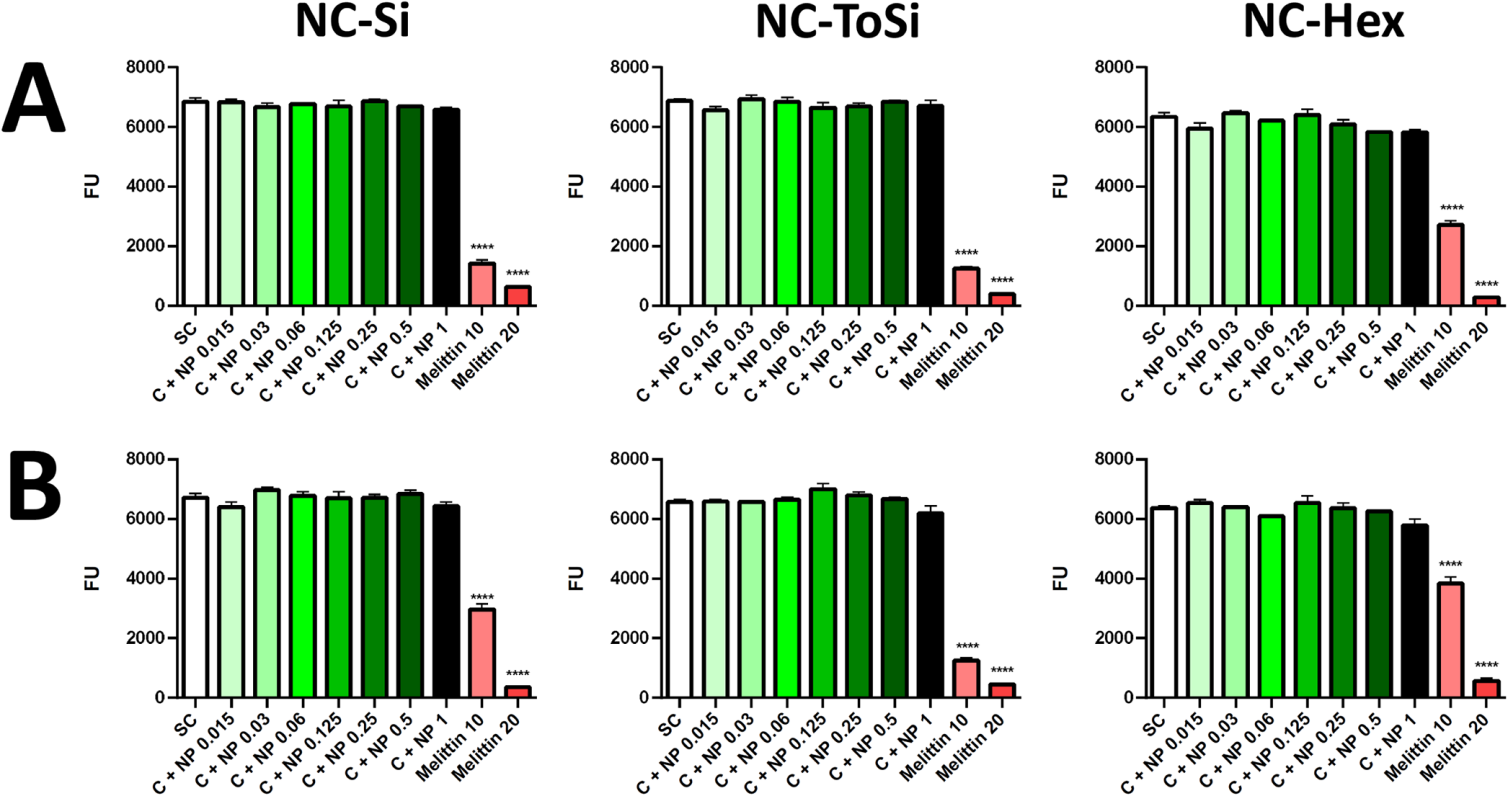
Caco-2 cell viability determined by resazurin assay following treatment with NC-Si, NC-ToSi, or NC-Hex. (A) Stirring at 300 rpm for 1 h, followed by 23 h of incubation. (B) Incubation without stirring for 24 h. SC (white): cells without nanochains. C + NP (green to black): cells with nanochains at different concentrations (0.015, 0.03, 0.06, 0.125, 0.25, 0.50, 1.00 mg/mL). Melittin (10 or 20 μg/mL) was used as a positive control (red). The significance of the differences was determined with one-way ANOVA with Dunnett’s posthoc test (***p < 0.001; ****p < 0.0001).

The nontoxicity of nanochains was further supported by microscopy. The cells showed no visible damage, and the monolayer remained intact regardless of the nanochain concentration or exposure to the rotating magnetic field. By contrast, melittin destroyed the integrity of the monolayer. These results demonstrate that the nanochains do not negatively affect Caco-2 cells, which maintain both their structural and functional integrity under the tested conditions (Figure 11).

**Figure 11:**
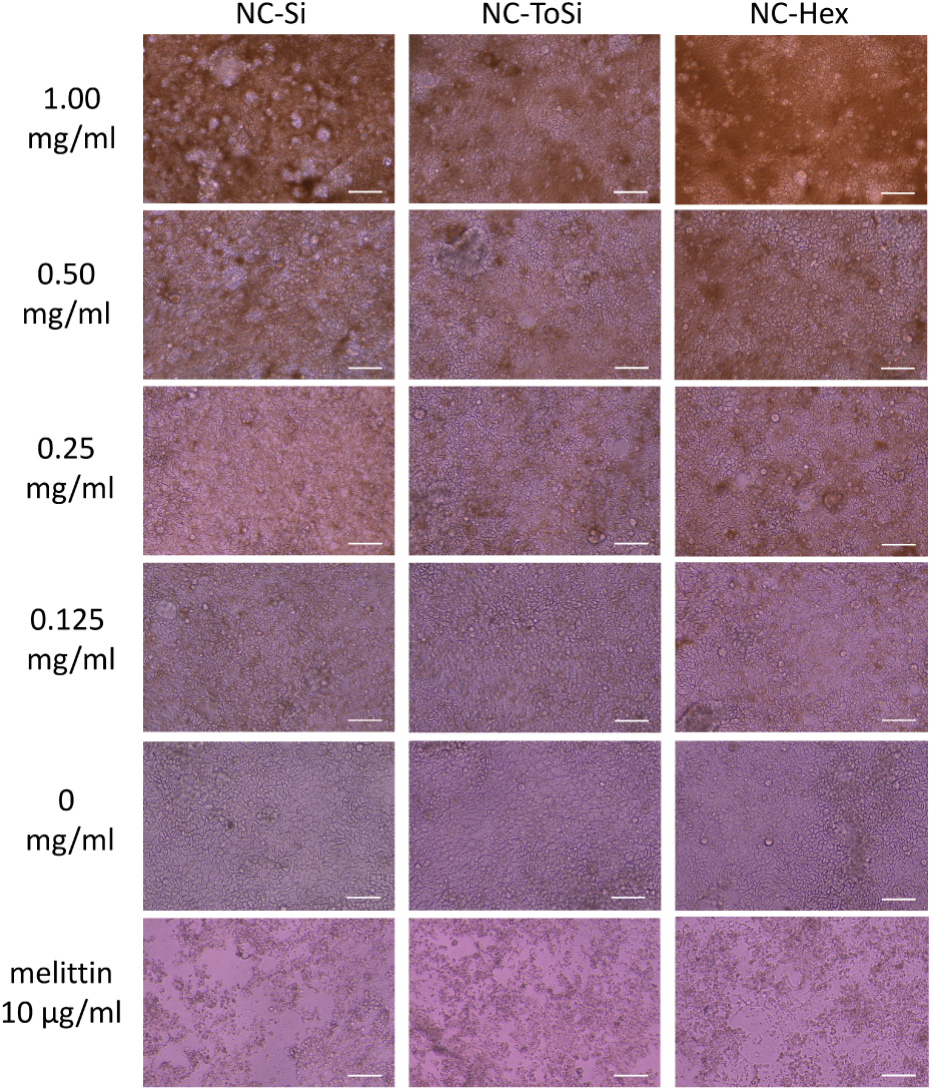
Integrity of Caco-2 cell monolayers observed under an optical microscope at 20× magnification. Monolayers were treated with NC-Si, NC-ToSi, or NC-Hex for 1 h using magnetic stirring at 300 rpm, followed by incubation for 23 h without stirring. The cells treated with higher concentrations of nanochains (0.125, 0.25, 0.50, and 1.00 mg/mL) are shown. Melittin was used as a positive control (10 μg/mL). Scale bar denotes 100 μm.

## Discussion

The present study describes anisotropic magnetic nanochains that are able to remove in excess of 90% of prewashed bacterial biofilm from a solid surface (microtiter plate). This is considered effective in terms of biomass removal, while in terms of viable cell removal, 99.9% or more would be preferred. However, the efficacy of such biofilm dispersal agents can be improved by combination therapy with antibiotics,^17,32^ which are *per se* known to be relatively ineffective in biofilm removal. As an alternative, biofilm dispersal could also be achieved with enzymatic approaches using glycoside hydrolases, which offer selectivity and low cell cytotoxicity; however, they are limited by lower stability and immunogenicity.^33^ Several nanosized antibiofilm technologies have also been suggested, including liposomes, nanoemulsions, polymers, and nanogels.^34^ Liposomes can improve antibiotic delivery and protection but can be rapidly cleared and potentially immunogenic.^34^ On the other hand, potential toxicity of inorganic nanochains should be considered. Here, their safety was preliminarily confirmed in an epithelial cell model.

Prewashed biofilms contain tightly attached cells and are more recalcitrant to mechanical treatment.^35^ Removal of prewashed biofilms thus supports the potential applicability of nanochains for cleaning biofilm-contaminated surfaces. In the present study, the biofilms of both Gram-positive and Gram-negative bacteria were removed; however, the efficacy depended on the species and, even more so, on the conditions tested. Although the roughness of the functional silica layer differed among NC-Si, NC-ToSi, and NC-Hex nanochains, it had limited or no significant impact on their efficacy. This may be due to the comparable sizes of all three types of nanochains. Varying the sizes of nanochains may be a topic of a separate study on alternative anisotropic particles in different length ranges. Nevertheless, smooth NC-Si demonstrated some species specificity, exhibiting lower efficacy in *E. coli* and higher efficacy in *L. lactis*.

Two magnetic stirring systems (characterized in Section S3) were compared for their efficacy in removing biofilms. Compared to the classic stirrer, the 96-well 2mag stirrer generates an approximately 20-fold weaker induced magnetic field under each well, providing localized, well-specified rotation with low (not detectable) magnetic field gradients. This applicator design hence maintains colloidal stability (particle dispersibility) by preventing the sedimentation of magnetic nanochains due to the magnetic aggregation of nanochains in a magnetic field gradient. By contrast, the classic lab magnetic stirrer relies on a single, large, rotating magnet under the entire microtiter plate and generates a stronger magnetic field as well as stronger magnetic field gradients (Figure S4B). This may lead to poor nanochain dispersibility and, thus, nanochain sedimentation due to aggregation triggered by relatively large magnetic field gradients, which may have caused variability within species and conditions. Expectedly, nanochain sedimentation was more of an issue with longer treatment times and lower stirring speeds, which decreased the ability of nanochains to effectively disrupt biofilms. The classic lab magnetic stirrer was generally better at removing biofilms, most probably due to a stronger magnetic field. This condition is related to more powerful nanochain rotational motion and, hence, larger magnetic torque and force transmission to biofilm components, resulting in more effective loosening of Gram-negative *E. coli* and *P. fragi* biofilms. However, the effectiveness of the stirrer could not be generalized, as it was less effective than the 96-well stirrer in the removal of Gram-positive *L. lactis* biofilm. It is known that *E. coli* DH5α forms intermediate biofilms,^36^ composed of layers of cells with a relatively low amount of EPS,^37^ which is in contrast to more pathogenic *E. coli* strains. The food-spoiling bacterium *P. fragi* often forms weak biofilms, which depend on medium composition, and is more abundant in EPS when grown in nutrient-limiting conditions and at low temperatures.^38^ Despite being a food starter, *L. lactis* can form unwanted biofilms on industrial equipment, which are, similar to *E. coli* composed of multiple layers of cells with low amounts of EPS,^39^ but they differ in surface charge, which is more negative in Gram-negative *E. coli*. Although the structural properties of the biofilms used in this study were relatively similar, minor differences may have contributed to the efficacy of their removal.

Treatment duration and stirring speed (rpm) played an important role in biofilm removal, but their effect was species- and stirrer-dependent. Maximal disruption of *E. coli* biofilm (>90%) was achieved using the classic lab magnetic stirrer for 60 min at 600 rpm. The same conditions were also effective in removing *L. lactis* biofilm with a 96-well stirrer, where longer treatment duration was more effective in general. In contrast, longer treatment duration (60 min) failed to increase the level of removal in some cases, such as *P. fragi* biofilm removal with the classic lab magnetic stirrer, where extended durations decreased efficacy. Optimal treatment duration and stirring speed, therefore, cannot be generalized and should be optimized on a case-by-case basis.

The reports on the use of anisotropic magnetic nanoparticles for biofilm removal have been relatively scarce. Larger magnetite microparticles (50 to 100 μm) have been used for magnetomechanical disruption of biofilm and ciprofloxacin delivery, decreasing the viability of *Staphylococcus aureus* and *Pseudomonas aeruginosa* by almost 80%.^40^ Nanoparticles (400 nm) based on halloysite nanotubes containing iron oxide cores removed up to 99% of *S. aureus* biofilm when combined with ampicillin.^41^ Anisotropic nanoparticles (200–300 nm) composed of iron oxide cores, coated with gold nanocrystals and fixed by polydopamine, caused over 50% biofilm removal by magnetomechanical action.^21^ Nanochains thus show comparable or superior magnetomechanical biofilm removal, along with high colloidal stability and superparamagnetic properties.

The complexity of the biofilm structure complicates the study of its composition and removal *in vitro*, and this is further exacerbated by the introduction of nanochains. Indirect methods, such as CFU counting, require quantitative removal of the biofilm, which is technically demanding. Direct methods, in contrast, typically rely on optical observation, which can be hindered by the presence of nanochains. Biofilm analysis can be enhanced by combining multiple approaches.^42^ Here, the monitoring of biofilm removal was complemented by the assessment of bacteria in the supernatant. For both approaches, two different methods were used. CFU counting is considered a standard method for biofilm studies^43^ and directly determines viable bacteria. Fluorescence measurements provide an alternative approach to quantifying biofilms^44^ and were used to assess the efficacy of removal and confirm magnetomechanical cell detachment. The two methods generally corresponded well across the experiments; however, some exceptions highlighted the limitations of fluorescence. The low fluorescence intensity of *P. fragi* was likely due to the weak promoter used, which decreased the sensitivity for detecting changes. Another factor affecting the match between the two methods was the nonhomogeneous nature of the biofilm samples. Detachment of larger fragments or uneven distribution of biofilm may have led to variability in fluorescence measurements. Additionally, the distribution of cells in wells was nonhomogeneous during biofilm measurement, as they were only present at the bottom and on the walls of the well. This affected the measurement of fluorescence, while CFU counts were relatively unaffected and provided a direct measure of viable cells. This, together with lower standard deviation, demonstrated that CFU counting is the most reliable method overall, particularly when fluorescence results were inconsistent or underestimated biofilm removal. In future work, additional insights from metabolic assays, EPS staining, and microscopy should be pursued.

In contrast to their effects on biofilms, the functionalized nanochains demonstrated no toxicity to the Caco-2 epithelial cell model, regardless of whether mechanical force was employed or not. This is in line with previous results on normal urothelial cells^45^ and suggests that the silica-coated iron-oxide nanochains prepared in this study neither leach toxic components nor cause mechanical damage to the cells or their monolayer. This is in contrast to copper-, zinc-, antimony-, manganese- and cobalt-oxide nanoparticles, which were found to be toxic in concentrations below 100 μg/mL in various cell lines.^46^ Iron oxide nanoparticle toxicity can be associated with ROS production^47^ and was shown to be size-, concentration-, and coating-dependent, with smaller sizes and lower concentrations exhibiting higher toxicity.^48,49^ Moreover, future studies should assess both the safety and efficacy of biofilm removal in *in vivo* models. However, this will require addressing several demanding challenges, including nanochain delivery, colloidal stability, and local application of rotational magnetic fields.

## Conclusion

We systematically assessed the efficacy of nanochains in the mechanical removal of prewashed attached biofilms by treating three bacterial species with three types of nanochains under various magnetic fields. The nanochains were able to remove all three bacterial biofilms with an efficacy of above or near 90% and exerted no negative effects on the Caco-2 cell monolayers.

## Supplementary Material



## Notes

Normalized VSM magnetization curves; zeta potential measurements; characterization of magnetic field distribution (PDF)

## #

Materials Research Laboratory, University of Nova Gorica, Vipavska 11c, 5720 Ajdovščina, Slovenia

## References

[1] Bamford N. C., MacPhee C. E., Stanley-Wall N. R. (2023). Microbial Primer: An introduction
to biofilms - what they are, why they form and their impact on built
and natural environments. Microbiology.

[2] Kostakioti M., Hadjifrangiskou M., Hultgren S. J. (2013). Bacterial biofilms: Development,
dispersal, and therapeutic strategies in the dawn of the postantibiotic
era. Cold Spring Harbor Perspect. Med..

[3] Flemming H. C., Wingender J. (2010). The biofilm
matrix. Nat. Rev.
Microbiol..

[4] Romling U., Balsalobre C. (2012). Biofilm infections, their resilience to therapy and
innovative treatment strategies. J. Int. Med..

[5] Donlan R. M. (2002). Biofilms:
Microbial life on surfaces. Emerg. Infect. Dis.

[6] Cassini A., Plachouras D., Eckmanns T., Abu Sin M., Blank H. P., Ducomble T., Haller S., Harder T., Klingeberg A., Sixtensson M. (2016). Burden of six healthcare-associated infections
on European population health: Estimating incidence-based disability-adjusted
life years through a population prevalence-based modelling study. PLoS Med..

[7] Gilbert P., Allison D., McBain A. (2002). Biofilms in
vitro and in vivo: Do
singular mechanisms imply cross-resistance?. J. Appl. Microbiol..

[8] Han, S.-K. Infection, debridement, and biofilm. In Innovations and Advances in Wound Healing; Springer, 2016; pp. 151–182.

[9] Mi G., Shi D., Wang M., Webster T. J. (2018). Reducing bacterial infections and
biofilm formation using nanoparticles and nanostructured antibacterial
surfaces. Adv. Healthcare Mater..

[10] Alumutairi L., Yu B., Filka M., Nayfach J., Kim M. H. (2020). Mild magnetic nanoparticle
hyperthermia enhances the susceptibility of *Staphylococcus
aureus* biofilm to antibiotics. Int.
J. Hyperther.

[11] Han C., Romero N., Fischer S., Dookran J., Berger A., Doiron A. L. (2017). Recent developments in the use of nanoparticles for
treatment of biofilms. Nanotechnol. Rev..

[12] Ibelli T., Templeton S., Levi-Polyachenko N. (2018). Progress on utilizing hyperthermia
for mitigating bacterial infections. Int. J.
Hyperther.

[13] Li L.-L., Yu P., Wang X., Yu S.-S., Mathieu J., Yu H.-Q., Alvarez P. J. (2017). Enhanced biofilm
penetration for microbial control
by polyvalent phages conjugated with magnetic colloidal nanoparticle
clusters (CNCs). Environ. Sci.: Nano.

[14] Quan K., Zhang Z., Chen H., Ren X., Ren Y., Peterson B. W., van der Mei H. C., Busscher H. J. (2019). Artificial channels
in an infectious biofilm created by magnetic nanoparticles enhanced
bacterial killing by antibiotics. Small.

[15] Quan K., Zhang Z., Ren Y., Busscher H. J., van der
Mei H. C., Peterson B. W. (2020). Homogeneous distribution of magnetic,
antimicrobial-carrying nanoparticles through an infectious biofilm
enhances biofilm-killing efficacy. ACS Biomater.
Sci. Eng..

[16] Kralj S., Makovec D. (2015). Magnetic assembly of
superparamagnetic iron oxide nanoparticle
clusters into nanochains and nanobundles. ACS
Nano.

[17] Kralj S., Da Silva C., Nemec S., Caf M., Fourquaux I., Rols M. P., Golzio M., Mertelj A., Kolosnjaj-Tabi J. (2025). Dynamically
assembling magnetic nanochains as new generation of swarm-type magneto-mechanical
nanorobots affecting biofilm integrity. Adv.
Healthcare Mater..

[18] Gorsak T., Drab M., Krizaj D., Jeran M., Genova J., Kralj S., Lisjak D., Kralj-Iglic V., Iglic A., Makovec D. (2020). Magneto-mechanical
actuation of barium-hexaferrite
nanoplatelets for the disruption of phospholipid membranes. J. Colloid Interface Sci..

[19] Yan J., Moreau A., Khodaparast S., Perazzo A., Feng J., Fei C., Mao S., Mukherjee S., Kosmrlj A., Wingreen N. S. (2018). Bacterial
biofilm material properties enable removal and transfer
by capillary peeling. Adv. Mater..

[20] Li J., Nickel R., Wu J., Lin F., van Lierop J., Liu S. (2019). A new tool to attack biofilms: Driving
magnetic iron-oxide nanoparticles
to disrupt the matrix. Nanoscale.

[21] Xu Y., Wang K., Zhu Y., Wang J., Ci D., Sang M., Fang Q., Deng H., Gong X., Leung K. C. (2023). Size-dependent
magnetomechanically enhanced
photothermal antibacterial effect of Fe(3)­O(4)@Au/PDA nanodurian. Dalton Trans.

[22] Nickel R., Kazemian M. R., Wroczynskyj Y., Liu S., van Lierop J. (2020). Exploiting
shape-selected iron oxide nanoparticles for the destruction of robust
bacterial biofilms - active transport of biocides via surface charge
and magnetic field control. Nanoscale.

[23] Pinto J. P., Zeyniyev A., Karsens H., Trip H., Lolkema J. S., Kuipers O. P., Kok J. (2011). pSEUDO, a
genetic integration standard
for *Lactococcus lactis*. Appl.
Environ. Microbiol..

[24] Stojanov S., Plavec T. V., Kristl J., Zupancic S., Berlec A. (2021). Engineering
of vaginal lactobacilli to express fluorescent proteins enables the
analysis of their mixture in nanofibers. Int.
J. Mol. Sci..

[25] Holo H., Nes I. F. (1995). Transformation of *Lactococcus* by electroporation. Methods Mol. Biol..

[26] Kovach M. E., Elzer P. H., Hill D. S., Robertson G. T., Farris M. A., Roop R. M., Peterson K. M. (1995). Four new derivatives
of the broad-host-range cloning vector pBBR1MCS, carrying different
antibiotic-resistance cassettes. Gene.

[27] Kralj S., Makovec D., Campelj S., Drofenik M. (2010). Producing ultra-thin
silica coatings on iron-oxide nanoparticles to improve their surface
reactivity. J. Magn. Magn. Mater..

[28] Tadic M., Kralj S., Jagodic M., Hanzel D., Makovec D. (2014). Magnetic properties
of novel superparamagnetic iron oxide nanoclusters and their peculiarity
under annealing treatment. Appl. Surf. Sci..

[29] Nemec S., Kralj S. (2021). A Versatile interfacial
coassembly method for fabrication of tunable
silica shells with radially aligned dual mesopores on diverse magnetic
core nanoparticles. ACS Appl. Mater. Interfaces.

[30] Kolosnjaj-Tabi J., Kralj S., Griseti E., Nemec S., Wilhelm C., Sangnier A. P., Bellard E., Fourquaux I., Golzio M., Rols M. P. (2019). Magnetic Silica-Coated Iron Oxide
Nanochains as Photothermal Agents, Disrupting the Extracellular Matrix,
and Eradicating Cancer Cells. Cancers.

[31] Kralj S., Makovec D. (2014). The chemically directed assembly
of nanoparticle clusters
from superparamagnetic iron-oxide nanoparticles. RSC Adv..

[32] Hawas S., Verderosa A. D., Totsika M. (2022). Combination therapies for biofilm
inhibition and eradication: A comparative review of laboratory and
preclinical studies. Front. Cell. Infect. Microbiol..

[33] Ramakrishnan R., Singh A. K., Singh S., Chakravortty D., Das D. (2022). Enzymatic dispersion of biofilms:
An emerging biocatalytic avenue
to combat biofilm-mediated microbial infections. J. Biol. Chem..

[34] Xie Y., Liu H., Teng Z., Ma J., Liu G. (2025). Nanomaterial-enabled
anti-biofilm strategies: New opportunities for treatment of bacterial
infections. Nanoscale.

[35] Hwang G., Klein M. I., Koo H. (2014). Analysis of
the mechanical stability
and surface detachment of mature *Streptococcus mutans* biofilms by applying a range of external shear forces. Biofouling.

[36] Wood T. K., Gonzalez Barrios A. F., Herzberg M., Lee J. (2006). Motility influences
biofilm architecture in Escherichia coli. Appl.
Microbiol. Biotechnol..

[37] Jayaraman A., Sun A. K., Wood T. K. (1998). Characterization
of axenic *Pseudomonas fragi* and Escherichia coli
biofilms that inhibit
corrosion of SAE 1018 steel. J. Appl. Microbiol..

[38] Briega I., Garde S., Sanchez C., Rodriguez-Minguez E., Picon A., Avila M. (2025). Evaluation of biofilm production
and antibiotic resistance/susceptibility profiles of Pseudomonas spp.
isolated from milk and dairy products. Foods.

[39] Mercier C., Durrieu C., Briandet R., Domakova E., Tremblay J., Buist G., Kulakauskas S. (2002). Positive role
of peptidoglycan breaks
in lactococcal biofilm formation. Mol. Microbiol..

[40] Bhuyan T., Simon A. T., Maity S., Singh A. K., Ghosh S. S., Bandyopadhyay D. (2020). Magnetotactic T-Budbots to Kill-n-Clean biofilms. ACS Appl. Mater. Interfaces.

[41] Mayorga-Martinez C. C., Zelenka J., Klima K., Kubanova M., Ruml T., Pumera M. (2023). Multimodal-driven magnetic
microrobots with enhanced
bactericidal activity for biofilm eradication and removal from titanium
mesh. Adv. Mater..

[42] Berlec A., Janez N., Sternisa M., Klancnik A., Sabotic J. (2021). Expression
of NanoLuc luciferase in Listeria innocua for development of biofilm
assay. Front. Microbiol..

[43] Thieme L., Hartung A., Tramm K., Graf J., Spott R., Makarewicz O., Pletz M. W. (2021). Adaptation of the Start-Growth-Time
Method for High-Throughput Biofilm Quantification. Front. Microbiol..

[44] Amador C. I., Stannius R. O., Roder H. L., Burmolle M. (2021). High-throughput screening
alternative to crystal violet biofilm assay combining fluorescence
quantification and imaging. J. Microbiol Methods.

[45] Potrc T., Kralj S., Nemec S., Kocbek P., Kreft M. E. (2023). The shape
anisotropy of magnetic nanoparticles: An approach to cell-type selective
and enhanced internalization. Nanoscale.

[46] Ivask A., Titma T., Visnapuu M., Vija H., Käkinen A., Sihtmäe M., Pokhrel S., Mädler L., Heinlaan M., Kisand V. (2015). Toxicity of 11 Metal
Oxide Nanoparticles to Three Mammalian Cell Types *In V.itro*. Curr. Top. Med. Chem..

[47] Sengul A. B., Asmatulu E. (2020). Toxicity of metal and
metal oxide nanoparticles: A
review. Environ. Chem. Lett..

[48] Feng Q. Y., Liu Y. P., Huang J., Chen K., Huang J. X., Xiao K. (2018). Uptake, distribution,
clearance, and toxicity of iron oxide nanoparticles
with different sizes and coatings. Sci. Rep..

[49] Naqvi S., Samim M., Abdin M. Z., Ahmed F. J., Maitra A. N., Prashant C. K., Dinda A. K. (2010). Concentration-dependent
toxicity
of iron oxide nanoparticles mediated by increased oxidative stress. Int. J. Nanomed..

