# Correction to “Magnetomechanical Detachment of Bacterial Biofilms Using Anisotropic Magnetic Iron Oxide Nanochains”

**DOI:** 10.1021/acsabm.5c02446

**Published:** 2026-01-02

**Authors:** Matija Šavli, Manca Černila, Maja Caf, Abida Zahirović, Nika Zaveršek, Sebastjan Nemec, Spase Stojanov, Anja Klančnik, Jerica Sabotič, Slavko Kralj, Aleš Berlec

An error was identified in the placement of Figures 8 and 9. During the revision process, these figures were inadvertently swapped. The corrected figures along with their captions are provided below.

**Figure 8:**
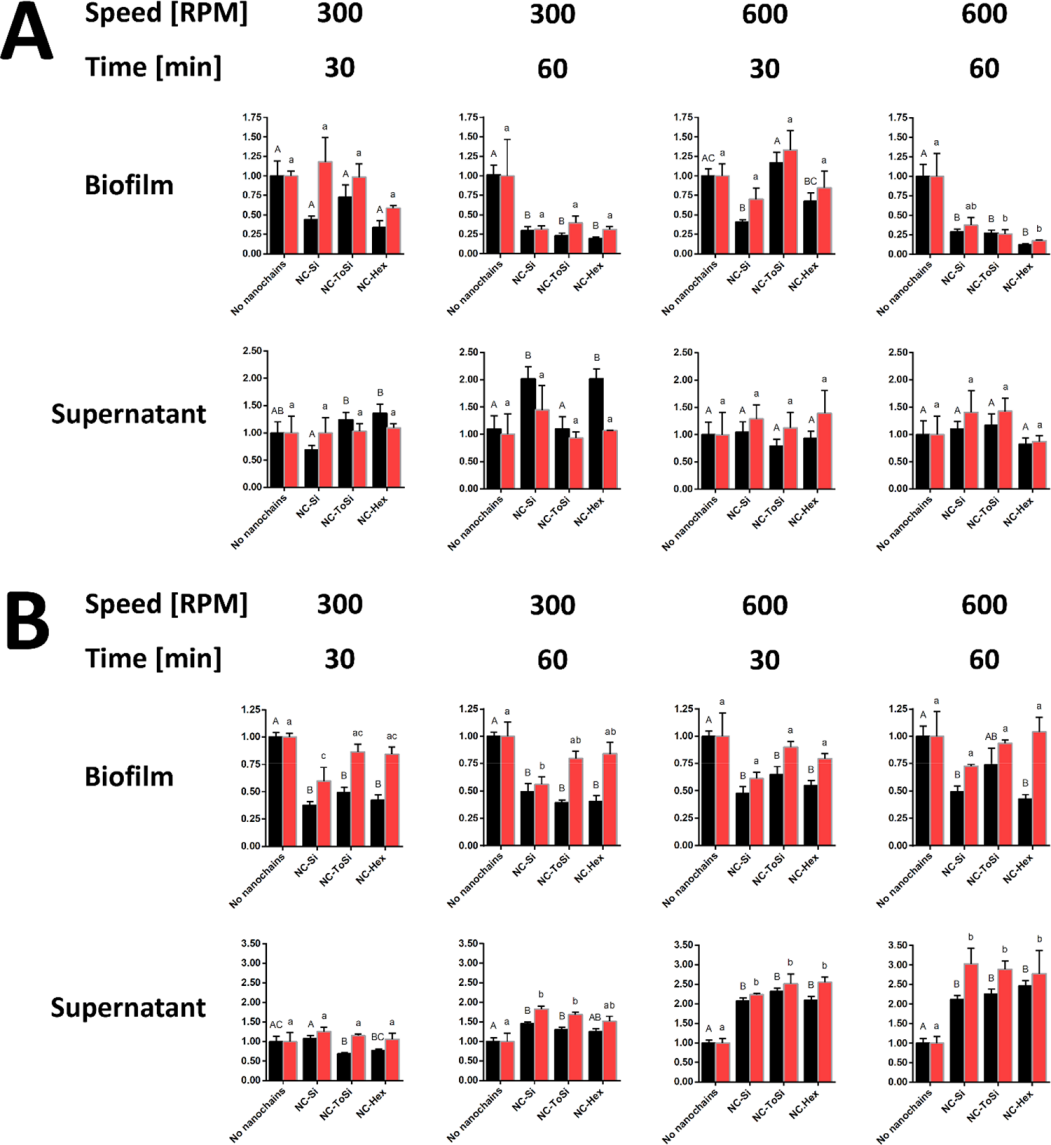
Treatment of *L. lactis* biofilms with functionalized nanochains (NC-Si, NC-ToSi, and NC-Hex). A 96-well stirrer (A) or classic lab magnetic stirrer (B) was used. Bacteria were quantified by CFU counting (black bars) and fluorescence measurement (FU, red bars) in both biofilms and supernatants after treatment. Data from CFU counting and fluorescence were normalized relative to the respective values obtained in biofilm samples to which no particles were added. The significance of the differences was determined with one-way ANOVA with Dunnett’s posthoc test. Differences were shown using a compact letter display; groups that do not share a letter are significantly different (*p* < 0.05).

**Figure 9:**
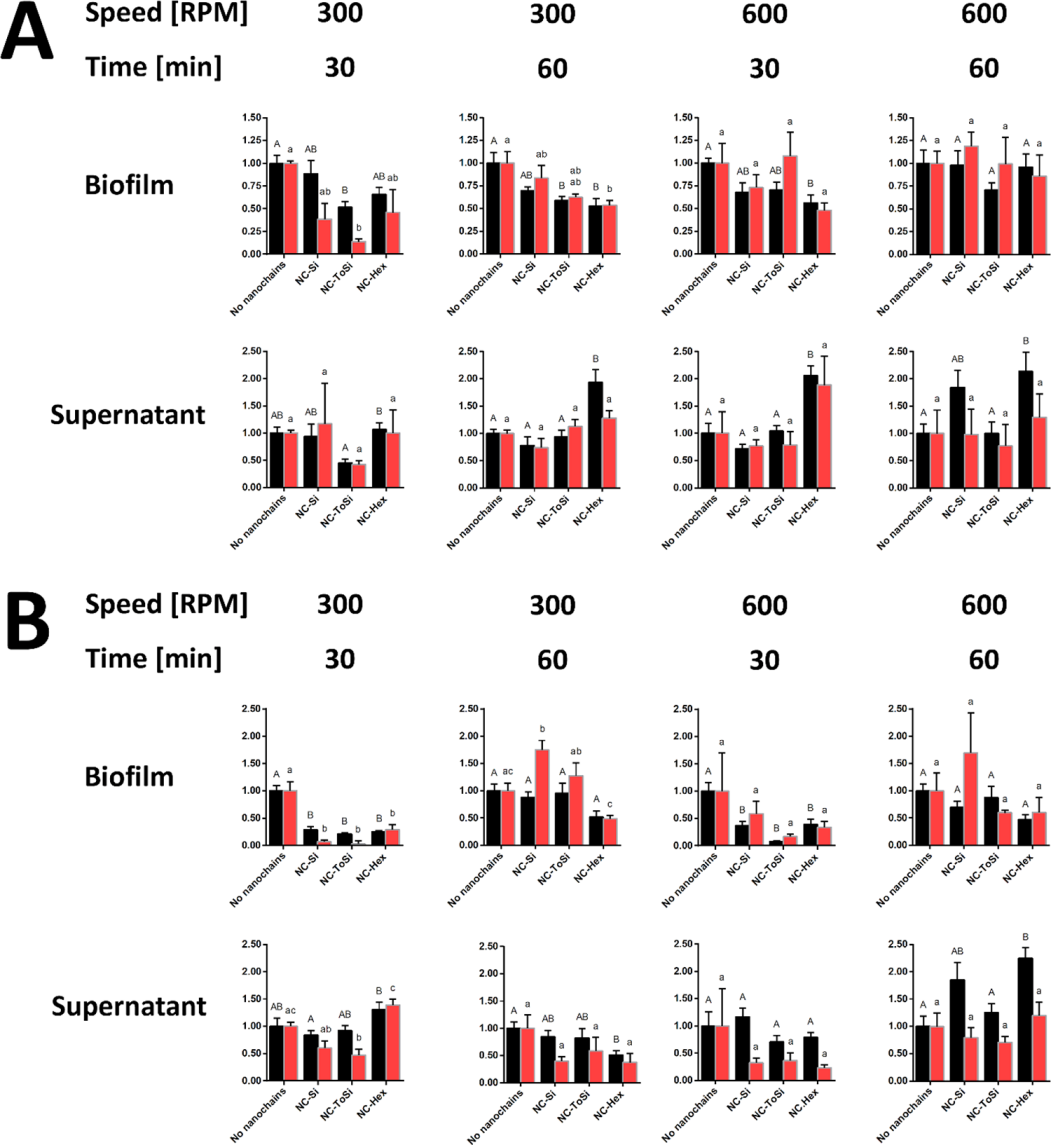
Treatment of *P. fragi* biofilms with functionalized nanochains (NC-Si, NC-ToSi, and NC-Hex). A 96-well stirrer (A) or classic lab magnetic stirrer (B) was used. Bacteria were quantified by CFU counting (black bars) and fluorescence measurement (FU, red bars) in both biofilms and supernatants after treatment. Data from CFU counting and fluorescence were normalized relative to the respective values obtained in biofilm samples to which no particles were added. The significance of the differences was determined with one-way ANOVA with Dunnett’s posthoc test. Differences were shown using a compact letter display; groups that do not share a letter are significantly different (*p* < 0.05).

