# Supplementary material for: Magnetomechanical Detachment of Bacterial Biofilms Using Anisotropic Magnetic Iron Oxide Nanochains
Source: ACS Appl Bio Mater. 2025 Sep 4;8(9):8059–71. doi: 10.1021/acsabm.5c01029 (PMC12442055; doi:10.1021/acsabm.5c01029)
Supplement: Supplementary file 1 [file mt5c01029_si_001.pdf]

Supporting Information

Magneto-mechanical detachment of bacterial biofilms using anisotropic magnetic iron oxide nanochains

Matija Šavli[1], Manca Černila[1], Maja Caf[2,3], Abida Zahirović[1], Nika Zaveršek[1], Sebastjan Nemec[2,#], Spase Stojanov[1], Anja Klančnik[4], Jerica Sabotič[1], Slavko Kralj[2,3,*], Aleš Berlec[1,3,*]

[1] Department of Biotechnology, Jožef Stefan Institute, Jamova 39, Ljubljana, Slovenia
[2] Department for Materials Synthesis, Jožef Stefan Institute, Jamova 39, Ljubljana, Slovenia
[3] Faculty of Pharmacy, University of Ljubljana, Aškerčeva 7, Ljubljana, Slovenia
[4] Department of Food Science and Technology, Biotechnical Faculty, Jamnikarjeva 101, University of Ljubljana, Ljubljana, Slovenia

[#] Present address: Materials Research Laboratory, University of Nova Gorica, Vipavska 11c, 5720 Ajdovščina, Slovenia

* Corresponding authors: ales.berlec@ijs.si, slavko.kralj@ijs.si

Section 1. Normalized VSM magnetization curves

In our previous work[1], we investigated the magnetic properties of magnetic nanochains in relation to their physical orientation. We demonstrated that these nanochains can exhibit either superparamagnetic or ferromagnetic behavior at room temperature, solely depending on their spatial arrangement. When the nanochains are randomly oriented, they display superparamagnetic behavior, whereas a predominantly parallel alignment results in ferromagnetic properties, with a coercive field (H_C) of approximately 100 Oe. This behavior can be attributed to shape anisotropy and strong inter-particle dipolar interactions. Zero-field-cooled (ZFC) and field-cooled (FC) magnetization curves further support these findings. For randomly distributed nanochains, the blocking temperature (T_B) was determined to be 123.6 K, while for parallelly aligned nanochains (with the magnetic field applied along the long axis, H = 100 Oe), T_B increased to 159.7 K. Notably, the ZFC curve for the aligned nanochains exhibited a broader peak, which is characteristic of strongly interacting magnetic nanoparticle assemblies. Similarly, Ehsan Sadat *et al.* reported that densely packed nanoparticle systems with enhanced dipolar interactions show significantly higher blocking temperatures compared to discrete nanoparticle systems.[2] In another work, the group led by Steven Connolly developed a magnetic assembly protocol to prepare superferromagnetic chains from initially superparamagnetic nanoparticles. Their study emphasized that close-packing of the nanoparticles leads to the emergence of strong local dipole fields, ultimately inducing ferromagnetic behavior in the assembled chains.[3]

The field dependence of magnetization, M(H), for randomly distributed nanochains exhibited no hysteresis at 250 K, with both coercivity (H_C) and remanent magnetization close to zero—characteristic of a superparamagnetic state. This behavior is consistent with expectations for maghemite nanoparticles with diameters around 10 nm. At lower temperatures (below the

blocking temperature, T_B), M(H) curves displayed non-zero coercivity, indicating blocking state of the nanoparticles in randomly distributed nanochains sample. As anticipated for iron oxide nanoparticles, H_C increases progressively with decreasing temperature below T_B. In contrast, the M(H) curves for parallelly aligned nanochains revealed clear ferromagnetic behavior, with a coercive field of $H_C = 98$ Oe, despite the constituent nanoparticles being individually superparamagnetic. This emergent ferromagnetism is attributed to collective magnetic behavior arising from the anisotropic assembly of nanoparticles, rather than from the intrinsic properties of the individual particles. These findings are in agreement with recent studies reporting that chains of spherical magnetite nanoparticles can exhibit high magnetic anisotropy as a result of their ordered, anisotropic arrangement.[4] In that work, magnetite nanoparticles with intrinsically low magneto-crystalline and shape anisotropy were compressed into dense assemblies that formed elongated chain-like bundles. These superstructures, with length scales of several hundred nanometers, demonstrated enhanced uniaxial magnetic anisotropy ($K_{eff} \sim 2.9 \times 10^5$ J/m³) and significant coercivity. Computational simulations indicated that these effects stem from strong interparticle dipolar interactions within the assembled chains.

Moreover, spherical nanoparticle clusters—the fundamental building blocks of nanochains—exhibit slightly lower magnetic susceptibility compared to the nanochains themselves, as demonstrated in our previous work.[5] Specifically, we observed a steeper slope in the magnetization curve for the nanochains, indicating a higher magnetic susceptibility, which can be attributed to their anisotropic shape. Given that both the nanochains and the nanoclusters were synthesized from the same primary iron oxide nanoparticles, this difference cannot be ascribed to variations in particle size, size distribution, crystallinity, defects, or internal strain. Instead, it is a direct consequence of their structural organization. Supporting this interpretation, Usov and Serebryakova[6] reported that the initial magnetic susceptibility of nanoparticle assemblies is strongly influenced by the packing density and interparticle magnetic dipolar interactions. Their theoretical analysis revealed that in weakly interacting systems, the equilibrium magnetization deviates significantly from the Langevin behavior at moderate and high magnetic fields due to the effect of magnetic anisotropy. In contrast, densely packed assemblies (nanoparticle cluster and nanochains) exhibiting strong magneto-dipolar coupling show a pronounced increase in initial susceptibility, depending on the packing density. Thus, deviations from Langevin susceptibility can be considered a hallmark of significant magneto-dipole interactions within the assembly, which also likely contribute to the enhanced susceptibility observed in our nanochains.

Additionally, the magnetic moments per nanoparticle within spherical nanoparticle clusters and nanochains were previously determined to be 20,526 μ_B and 20,767 μ_B, respectively.[7] The slightly higher magnetic moment observed for nanochains can be attributed to their structural anisotropy, which facilitates more efficient alignment of the magnetic moments along the applied field direction. This shape anisotropy leads to enhanced magnetic susceptibility at low magnetic fields, thereby contributing to the overall easier magnetization of the anisotropic nanochain structures compared to their spherical counterparts which is highly relevant for biofilm removal process.

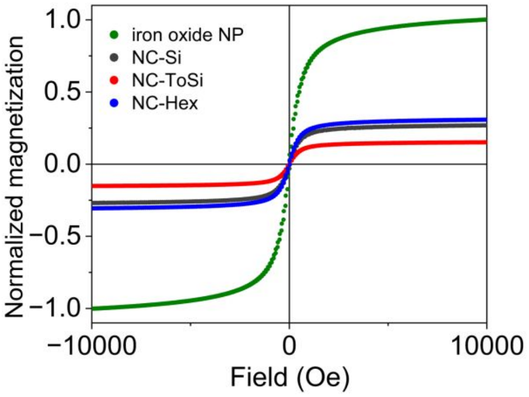

Figure S1: Room-temperature measurements of the magnetization as a function of magnetic field for all three types of functionalized nanochains (NC-Si, NC-ToSi, NC-Hex) normalized to initial iron oxide nanoparticles (γ-Fe_2O_3).

Section 2. Zeta potential measurements

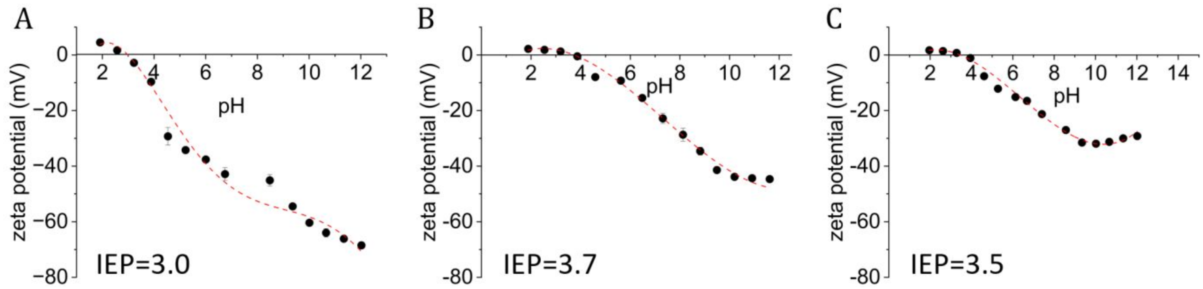

Figure S2: Zeta potential measurements for silica-coated nanochains before any functionalization. Non-functionalized nanochains with compact silica (Si) (A), non-functionalized nanochains with ToSi rough silica (B), and non-functionalized nanochains with Hex rough silica (C).

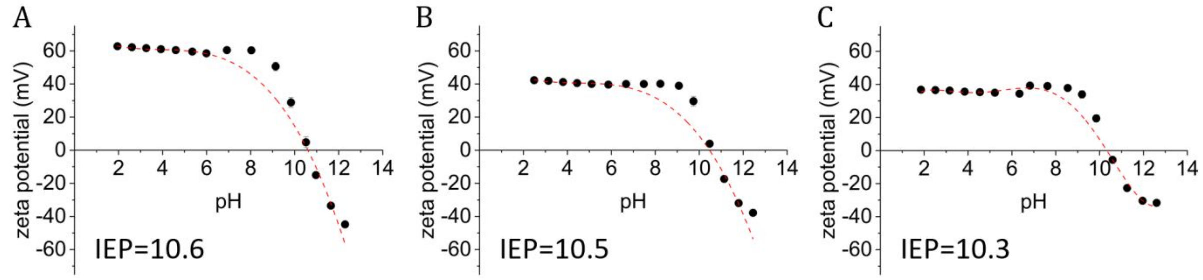

Figure S3: Zeta potential measurements for APTES-functionalized nanochains as an intermediate stage in the process. APTES-functionalized nanochains with compact silica (Si) (A), APTES-functionalized nanochains with ToSi rough silica (B), and APTES-functionalized nanochains with Hex rough silica (C).

Section 3. Characterization of magnetic field distribution in two setup configurations

The experiments were conducted using a 96-well plate. The magnetic field strength was measured at the bottom of each individual well. Measurements were performed using a portable magnetometer (LakeShore Model 460; 3-Channel Gaussmeter, Serial Number 46800, USA). For the 2mag induction stirrer setup (Figure S4, A), no significant variation in magnetic field strength was observed within a 3 mm shift along the z-axis. In contrast, for the Domel Rotamix SHP-10 device (Figure S4, B), a decrease of approximately 1.5 mT was

recorded (from 1.3 mT to 1.8 mT depending on the position), corresponding to an average magnetic field gradient of approximately 0.5 T/m.

A

2mag 96 well induction mixer

P = 100 % 600 RPM

[mT]	1	2	3	4	5	6	7	8	9	10	11	12
A	2,7	2,5	2,5	2,7	2,4	2,7	2,4	2,4	2,3	2,6	2,3	2,7
B	2,6	1,7	2,0	1,9	2,1	1,9	2,0	1,7	1,9	1,9	1,9	2,0
C	2,3	2,1	2,1	2,2	2,2	2,6	2,1	2,3	2,0	2,2	1,9	2,5
D	2,4	2,0	2,1	1,9	2,2	1,9	2,2	1,9	2,0	1,8	1,9	2,1
E	2,5	2,0	1,9	2,1	2,0	2,1	2,0	2,1	1,9	2,0	1,8	2,3
F	2,4	2,1	2,1	2,0	2,1	2,0	2,0	2,2	1,9	1,9	1,9	2,3
G	2,4	1,9	1,9	1,9	1,9	2,1	2,1	2,0	2,0	1,9	1,7	2,3
H	2,3	2,3	2,3	2,3	2,4	2,5	2,5	2,5	2,4	2,4	2,3	2,5

**All**	
min	1,7
max	2,7
average	2,2

B

Domel Rotamix SHP-10

600 RPM

[mT]	1	2	3	4	5	6	7	8	9	10	11	12
A	8,5	16,1	24,1	34,1	42,1	45,2	45,1	41	32,1	20,8	13,2	8,1
B	13,8	22,1	33,1	44,6	49,7	48,7	47,5	48,5	43,9	29,6	17,4	10,2
C	16,2	26,3	38,5	48,6	48,2	46,1	45,4	46	46,3	37,6	22,3	12,5
D	18,1	28,3	41,3	48,7	47	45,3	44,8	45,7	46,9	41,6	25,4	14
E	17,2	28	41,6	49,2	48,1	45,6	45,8	46,5	48,4	41	24,6	13,5
F	15,8	25,3	37,6	47	49,4	48,2	48,2	48,9	48,2	37,3	22,1	11,9
G	12,8	20,3	30,9	41,7	47,2	48,9	48,6	47,4	41,9	29,6	17,6	9,6
H	10,1	15,1	22,4	32,2	37,2	39,3	38,3	35,4	30,2	20,7	12,8	7,6

**All**	
min	7,6
max	49,7
average	33,4
**Experimental part (encircled)**	
min	41,7
max	49,7
average	47,0

Figure S4: Magnetic field strengths under individual wells of 96-well plate positioned on the 96-well 2mag induction plate stirrer (A) or classic stirrer – Domel Rotamix SHP-10 (B).

References

(1) Tadic, M.; Kralj, S.; Lalatonne, Y.; Motte, L. Iron oxide nanochains coated with silica: Synthesis, surface effects and magnetic properties. *Applied Surface Science* **2019**, *476*, 641-646. DOI: 10.1016/j.apsusc.2019.01.098.

(2) Sadat, M. E.; Bud'ko, S. L.; Ewing, R. C.; Xu, H.; Pauletti, G. M.; Mast, D. B.; Shi, D. L. Effect of dipole interactions on blocking temperature and relaxation dynamics of superparamagnetic Iron-Oxide (Fe_3O_4) nanoparticle systems. *Materials* **2023**, *16* (2). DOI: Artn 496
10.3390/Ma16020496.

(3) Tay, Z. W.; Savliwala, S.; Hensley, D. W.; Fung, K. L. B.; Colson, C.; Fellows, B. D.; Zhou, X. Y.; Huynh, Q.; Lu, Y.; Zheng, B.; et al. Superferromagnetic nanoparticles enable order-of-magnitude resolution & sensitivity gain in magnetic particle imaging. *Small Methods* **2021**, *5* (11). DOI: Artn 2100796
10.1002/Smtd.202100796.

(4) Mohapatra, J.; Joshi, P.; Abbas, H.; Gusenbauer, M.; Bian, K. F.; Lu, P.; Fan, H. Y.; Schrefl, T.; Liu, J. P. Superstructure magnetic anisotropy in Fe_3O_4 nanoparticle chains. *Nature communications* **2025**, *16* (1). DOI: 10.1038/S41467-025-60888-X.

(5) Kopanja, L.; Tadic, M.; Kralj, S.; Zunic, J. Shape and aspect ratio analysis of anisotropic magnetic nanochains based on TEM micrographs. *Ceram Int* **2018**, *44* (11), 12340-12351. DOI: 10.1016/j.ceramint.2018.04.021.

(6) Usov, N. A.; Serebryakova, O. N. Equilibrium properties of assembly of interacting superparamagnetic nanoparticles. *Scientific reports* **2020**, *10* (1). DOI: 10.1038/S41598-020-70711-W.

(7) Tadic, M.; Kralj, S.; Kopanja, L. Synthesis, particle shape characterization, magnetic properties and surface modification of superparamagnetic iron oxide nanochains. *Mater Charact* **2019**, *148*, 123-133. DOI: 10.1016/j.matchar.2018.12.014.
